# Sox2 controls Schwann cell self-organization through fibronectin fibrillogenesis

**DOI:** 10.1038/s41598-019-56877-y

**Published:** 2020-02-06

**Authors:** Elen Torres-Mejía, Dietrich Trümbach, Charlotte Kleeberger, Ulf Dornseifer, Tanja Orschmann, Theresa Bäcker, Jara Kerstin Brenke, Kamyar Hadian, Wolfgang Wurst, Hernán López-Schier, Sabrina C. Desbordes

**Affiliations:** 1 Stem Cells in Neural Development and Disease group, Helmholtz Zentrum München, German Research Center for Environmental Health, 85764 Munich-Neuherberg, Germany; 2 Research Unit Sensory Biology and Organogenesis, Helmholtz Zentrum München, German Research Center for Environmental Health, 85764 Munich-Neuherberg, Germany; 3 Institute of Developmental Genetics, Helmholtz Zentrum München, German Research Center for Environmental Health, 85764 Munich-Neuherberg, Germany; 4 Assay Development and Screening Platform, Helmholtz Zentrum München, German Research Center for Environmental Health, 85764 Munich-Neuherberg, Germany; 5 Stem Cell Based-Assay Development Platform (SCADEV), Helmholtz Zentrum München, German Research Center for Environmental Health, 85764 Munich-Neuherberg, Germany; 6 Chair of Developmental Genetics, Technische Universität München-Weihenstephan, 85350 Freising-Weihenstephan, Germany; 7 German Center for Neurodegenerative Diseases (DZNE), 81377 Munich, Germany; 8 Department of Plastic, Reconstructive, Hand and Burn Surgery, Academic Hospital Bogenhausen, Munich, 81925 Germany; 9 Present Address: ISAR Bioscience GmbH, Institute for Stem Cell & Applied Regenerative Medicine Research, Semmelweisstr. 5, 82152 Munich, Germany

**Keywords:** Collective cell migration, Extracellular matrix

## Abstract

The extracellular matrix is known to modulate cell adhesion and migration during tissue regeneration. However, the molecular mechanisms that fine-tune cells to extra-cellular matrix dynamics during regeneration of the peripheral nervous system remain poorly understood. Using the RSC96 Schwann cell line, we show that Sox2 directly controls fibronectin fibrillogenesis in Schwann cells in culture, to provide a highly oriented fibronectin matrix, which supports their organization and directional migration. We demonstrate that Sox2 regulates Schwann cell behaviour through the upregulation of multiple extracellular matrix and migration genes as well as the formation of focal adhesions during cell movement. We find that mouse primary sensory neurons and human induced pluripotent stem cell-derived motoneurons require the Sox2-dependent fibronectin matrix in order to migrate along the oriented Schwann cells. Direct loss of fibronectin in Schwann cells impairs their directional migration affecting the alignment of the axons *in vitro*. Furthermore, we show that Sox2 and fibronectin are co-expressed in proregenerative Schwann cells *in vivo* in a time-dependent manner during sciatic nerve regeneration. Taken together, our results provide new insights into the mechanisms by which Schwann cells regulate their own extracellular microenvironment in a Sox2-dependent manner to ensure the proper migration of neurons.

## Introduction

The precise control of local extracellular microenvironmental remodelling contributes to appropriate cell movement throughout important processes such as tissue repair^1,2^. In the peripheral nervous system (PNS), Schwann cells, the main glial cells of the PNS, have the ability to produce extracellular matrix proteins, such as glycoproteins, proteoglycans and non-proteoglycan polysaccharides, forming the basal lamina^3^. During PNS regeneration, these proteins contribute to the formation of a permissive microenvironment that facilitates glial cell proliferation, migration and axon regeneration^3^. After PNS injury, Schwann cells reprogram to a proregenerative state^4^, proliferate and migrate to form the bands of Büngner, which are cellular channels made of glial basal lamina that guide the axons to their original targets^5^. How Schwann cells regulate their surrounding ECM and how much this contributes to their own behaviour and later axonal migration is not completely understood.

In contrast to the central nervous system, neurons from the PNS have the capacity to regrow after injury; however, this capacity can be compromised depending on the severity of the injury and the level of damage of the endoneurial tubes. After a crush injury, where the axon is affected but the basal lamina and connective tissue remain, the axon follows the original path and reconnects to the target, which leads to functional recovery^5^. After an axotomy, where the structure of the nerve is damaged and connective tissue and basal lamina are interrupted, axonal regeneration and functional recovery are less efficient due to axon atrophy or incorrect target reinnervation^5^. Understanding the molecular cues that guide the axons during the process of regeneration will allow us to improve the design of artificial conduits that help axons to regrow.

Sox2 is a regulator of the proregenerative Schwann cell behaviour^6^. It belongs to the family of high-mobility group transcription factors and it is well known for its role in pluripotent stem cell maintenance and as a reprogramming factor of differentiated cells to induced pluripotent stem cells, in combination with other reprogramming transcription factors^7^. During regeneration of the PNS, Sox2 expression in Schwann cells is important for their maintenance in a dedifferentiated stage as well as their sorting, mediated by the cell-cell adhesion protein N-cadherin^6^. Sox2 expression is induced by the endoneurial fibroblasts through ephrin-B/EphB2 signalling, a family of receptor tyrosine kinases^6^. Fibroblasts express ephrin-B ligand and bind to the EphB2 receptor in Schwann cells, which induces a relocalization of N-cadherin, from the cytoplasm to the membrane promoting Schwann cell-cell adhesion^6^.

Cadherin-mediated cell-cell adhesion is an important mechanism by which multicellular structures form during morphogenesis and regeneration, acting in coordination with integrin-mediated cell-ECM adhesion^8^. It is known that integrin-based cell-ECM adhesion also plays a critical role in Schwann cells’ and neurons’ migration^9^. However, whether Sox2 directly regulates Schwann cell behaviour through integrin-mediated cell-ECM adhesion is unexplored. A key molecule involved in integrin-mediated cell-ECM adhesion is Fibronectin (FN), which is required for proper Schwann cell migration and axonal regrowth^10–14^. After PNS injury, FN fibrillogenesis, a process by which FN assembles into fibrils, is increased at the injury site, supporting cellular adhesion and inducing the migration and proliferation of the Schwann cells, which in turn favours axonal growth^15,16^.

Here, we established a 2D *in vitro* system to study how Sox2-expressing Schwann cells modulate their own ECM microenvironment to promote guided axonal growth. We identified Sox2 as a direct regulator of FN expression and fibrillogenesis in the rat Schwann cell line RSC96, enabling their organization and directional migration, guiding mouse primary sensory neurons independently of fibroblasts. Furthermore, we show that hiPSC-derived motoneurons can also be guided by Sox2-expressing RSC96 Schwann cells. Finally, we reveal co-expression of the FN splice variant containing the EIIIA domain and Sox2 in Schwann cells *in vivo* during sciatic nerve regeneration after axotomy in rats. Taken together, our results suggest a novel role of Sox2 as a regulator of cell-ECM adhesion through FN fibrillogenesis for proper organization of Schwann cells and neurons.

## Results

### Sox2 triggers directional schwann cell migration

To understand the crosstalk between the ECM and pro-regenerative Schwann cells mediated by Sox2, we established an *in vitro* model system using the rat Schwann cell line RSC96 (SC^wt^). The SC^wt^ is highly proliferative and, unlike other Schwann cell lines, was not generated from a tumour^17–20^. SC^wt^ is a spontaneously immortalized cell line from long-term culture of rat primary Schwann cells and it has a non-myelinating phenotype^17,18^. Furthermore, this cell line expresses almost undetectable protein levels of Sox2, which make it a suitable model to study the role of Sox2 expression in cell behaviour.

**Figure 1:**
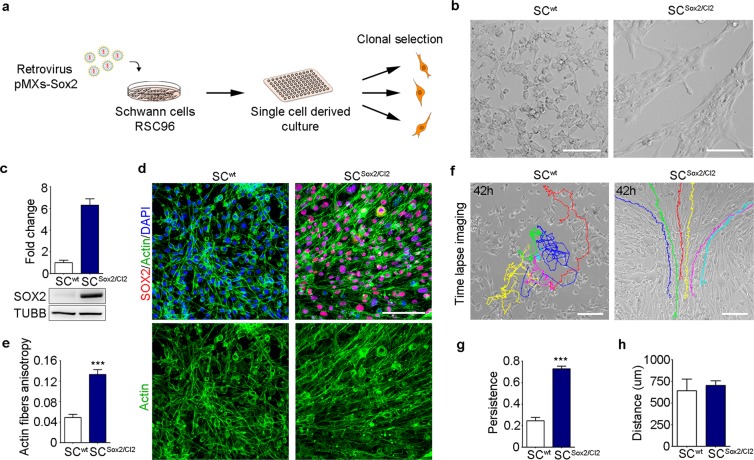
Sox2 overexpression induces Schwann cell adhesion and directional migration. (**a**) Schematic representation of the protocol used to derive Sox2-positive clones from the rat Schwann cell line RSC96. (**b**) Bright-field images of SC^wt^ and Sox2-positive clone SC^Sox2/Cl2^ after Sox2 transduction. Scale bar, 100 μm. (**c**) Western blot analysis of total SOX2 protein levels in the whole lysate of SC^wt^ and SC^Sox2/Cl2^. TUBB was used as a loading control (N = 3). (**d**) Representative immunostaining confocal images of SOX2 (red) and Actin fibres (green) of SC^wt^ SC^Sox2/Cl2^ in FBS-supplemented medium. Nuclei were counterstained with DAPI (blue). Scale bar, 100 μm. (**e**) Quantification of actin fibre anisotropy by actin staining of SC and SC^Sox2/Cl2^ (N = 5). (**f**) Representative time-lapse images from Supplementary Movies 1, 2 of SC^wt^ and SC^Sox2/Cl2^. Color lines show single-cell tracks of the Schwann cells for 42 hours. Scale bar, 100 μm. (**g**,**h**) Quantification of persistence and travelled distance of SC^wt^ and SC^Sox2/Cl2^, quantification was performed considering only 100 frames per cell (n = 10 cells). Graphs show mean value ± s.e.m, ***p < 0.0005.

We overexpressed Sox2 via retroviral infection and selected clones with different Sox2 expression levels (Clone (Cl) 1 to 6, Fig. 1a). We used cells that were transduced with an empty vector as a control clone (Cl0). Sox2 over-expression was confirmed by quantitative real time RT-qPCR (Supplementary Fig. 1a). In agreement with a previous study^6^, we observed a change in the behaviour of the Sox2-overexpressing cells, characterized by increased clustering as shown in Fig. 1b for one example clone, SC^Sox2/Cl2^ (Fig. 1c and Supplementary Fig. 1a; full-length blots are presented in Supplementary Fig. 1b).

We next analysed mRNA levels of markers of different states of Schwann cells (Supplementary Fig. 1c–f). We evaluated *c-Jun*, a key transcription factor which induces and maintains Schwann cells in a proregenerative phenotype^21^, *Krox 20*, a transcription factor that induces myelination^22^, *Krox 24* a transcriptions factor that maintains Schwann cells in a non-myelinated and proliferative stage^22^, and G*fap*, the glial fibrillary acidic protein, up-regulated in Schwann cells during PNS regeneration^23^. We observed that mRNA levels of *c-Jun, Krox20*, and *Krox24* did not change after Sox2 overexpression (Supplementary Fig. 1c–e). In contrast, *Gfap* mRNA and protein levels were significantly higher in Sox2-positive cells compared to controls, mimicking a regenerative situation (Supplementary Fig. 1f,g).

We next labelled actin filaments in SC^wt^ and the clones using the bicyclic peptide Phalloidin, to study cell-to-cell alignment after Sox2 overexpression. We evaluated cell organization by measuring the alignment of actin fibres between cells (anisotropy of actin fibres) using the FibrillTool (ImageJ plug-in)^24^. Anisotropy quantification was performed analysing different areas of a defined size (Supplementary Fig. 1h). Values close to one (high anisotropy) imply that cells are parallel to each other. We observed that SC^wt^ and SC^Cl0^ presented a low actin filament anisotropy while overexpression of Sox2 in these cells significantly increased the anisotropy, SC^wt^: 0.04911 ± 0.006136 vs SC^Sox2/Cl2^: 0.1331 ± 0.00942 (Fig. 1d,e and Supplementary Fig. 1i,j).

To better decipher the cell behaviour leading to cell alignment observed in SC^Sox2^, we performed time-lapse imaging in SC^wt^ and SC^Sox2/Cl2^. The tracking of cell behaviour showed that SC^wt^ move randomly, whereas SC^Sox2/Cl2^ follow a defined path (Fig. 1f and Supplementary Movies 1, 2). We also observed that overexpression of Sox2 increased the substrate coverage by the SC^Sox2/Cl2^ (Supplementary Movies 3, 4). In addition, the capacity to maintain directionality (persistence) was significantly increased in SC^Sox2/Cl2^ (3-fold change), whereas the travelled distance was not different between wild-type and Sox2-expressing cells (Fig. 1g,h). These results show that Sox2 overexpression promotes directional migration in Schwann cells *in vitro*.

### Cell adhesion- and ECM-related genes are up-regulated in Sox2 overexpressing Schwann cells

**Figure 2:**
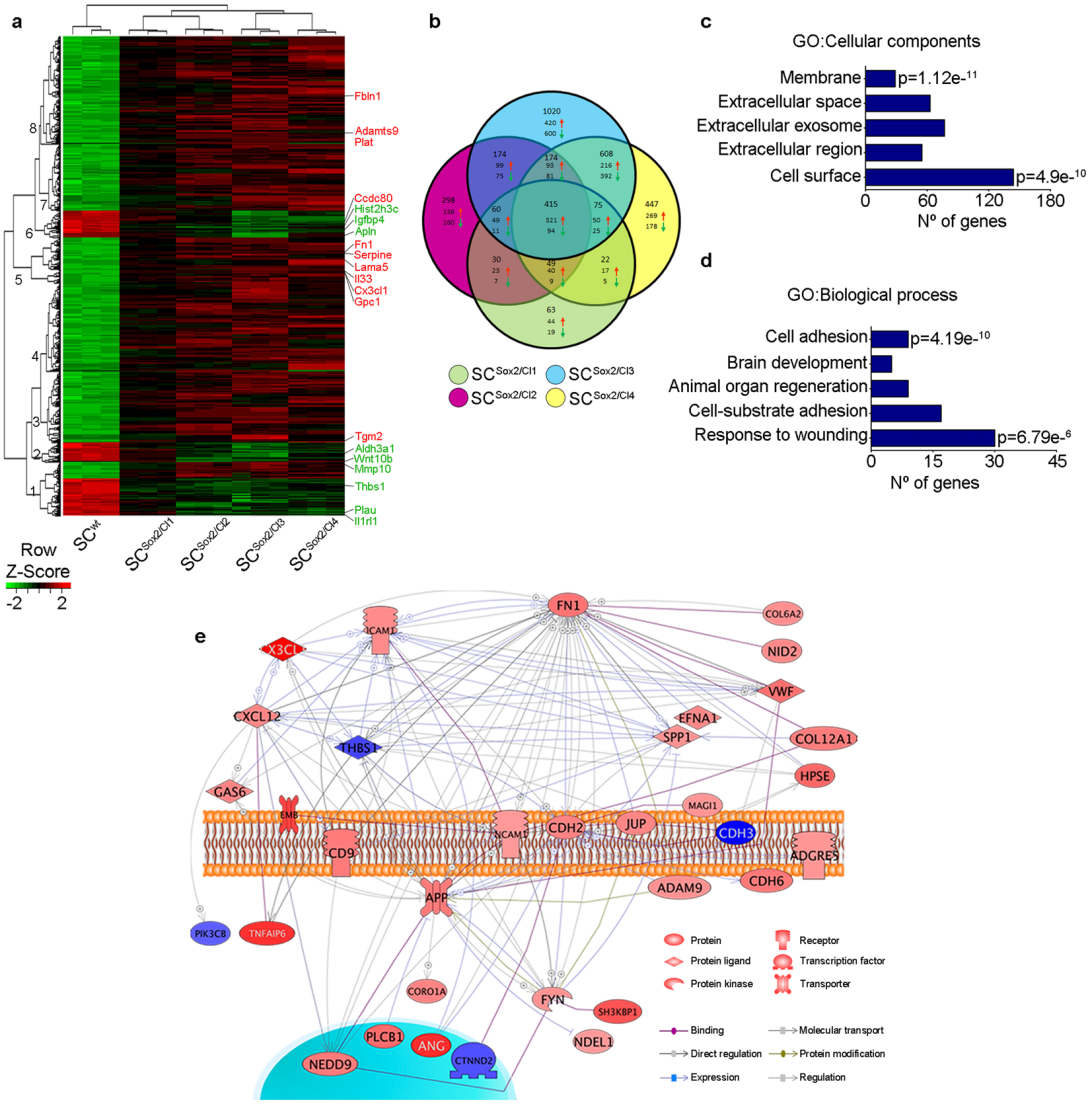
Cell adhesion- and ECM related-genes are significantly up-regulated upon Sox2 overexpression in Schwann cells. (**a**) Heat map of the normalized overlapping genes differentially expressed in four selected Sox2-positive Schwann cell clones (SC^Sox2/Cl1^, SC^Sox2/Cl2^, SC^Sox2/Cl3^, SC^Sox2/Cl4^) compared to the wild type (SC^wt^). The top significantly up- and downregulated ECM-related genes are highlighted. (**b**) Venn diagram of differentially regulated genes in Sox2 clones. (**c**,**d**) Gene ontology (GO) enrichment analysis for biological process and cellular components for the up-regulated overlapping genes in four Sox2-positive clones (SC^Sox2/Cl1^, SC^Sox2/Cl2^, SC^Sox2/Cl3^, SC^Sox2/Cl4^). (**e**) Literature-derived network of ECM related genes involved in cell adhesion and migration showing *Fn1* as a highly interconnected gene within the network. Probes with a p-adjusted < 0.002 and 2 < fold change (FC) <−2 were considered for the analysis.

To identify ECM-related genes and pathways involved in Schwann-cell directional migration induced by Sox2, we performed a microarray analysis of total RNA extracted from SC clones (Cl1 to Cl4) expressing different levels of Sox2. We found a total of 714 (Cl1), 1200 (Cl2), 1790 (Cl3) and 2526 (Cl4) genes differentially regulated by using probes with corrected p-values < 0.002 and fold change (FC) cut-offs of >2 for upregulated genes and <−2 for downregulated genes. We created a hierarchical clustering represented with a heat map, where we identified the top 20 ECM-related genes that were differentially regulated (Fig. 2a). We also derived eight clusters to study subtrees of the row dendrogram representing similar gene expression profiles as a result of the hierarchical clustering according to biological function (Supplementary Dataset 1). We then performed enrichment analysis on the 415 common genes (Fig. 2b) that were significantly up-regulated in the four SC^Sox2^ clones compared to wild-type cells. We found that ECM components and cell adhesion were the most significantly represented categories looking at the gene ontology categories ‘cellular components’ and ‘biological processes’ (Fig. 2c,d). Top genes involved in ECM and cell-cell adhesion were validated by real-time RT-qPCR (Supplementary Fig. 2a–j). To identify key Sox2-regulated ECM genes in SC^Sox2^ clones, we took the 43 differentially expressed ECM genes related to cell adhesion and migration and created a literature-derived network based on text mining for direct interactions. As represented in Fig. 2e, *Fn1* was identified as a highly interconnected gene, suggesting that it could be a key effector gene downstream of Sox2. Taken together, these results suggest that overexpression of Sox2 in the Schwann cell line RSC96 leads to ECM and migration gene upregulation, with FN as potential key regulator.

### Sox2 induces FN fibrillogenesis and focal adhesion in Schwann cells

Expression of FN and the accumulation of FN fibrils is important during major cellular processes. To evaluate FN expression in Schwann cells, we changed to a FN-free medium condition. We substituted foetal bovine serum, which contains high levels of FN, by a knock-out serum replacement, with undetectable FN levels, evaluated by western blot (Supplementary Fig. 3a). We first evaluated cell proliferation of SC^wt^ and SC^Sox2/Cl2^ in the maintenance medium (NM) and in the medium containing the knock-out serum replacement (KSRM) by counting the number of cells every 24 hour during 3 days. At day one and two, there were no significant differences between the cell lines or between the same cell line cultured in NM or KSRM. At day three, the number of SC^wt^ was lower in KSRM compared to NM (p = 0.0006), and we did not find significant differences in SC^Sox2/Cl2^ cultured in NM and KSRM. When we compared cell proliferation after three days between SC^wt^ and SC^Sox2/Cl2^ in the NM, we found that the cell number of SC^wt^ were significantly higher than the cell number of SC^Sox2/Cl2^ (p = 0.0162). However, no significant differences were found between SC^wt^ and SC^Sox2/Cl2^ cultured in KSRM. In summary, we found that when grown in knockout serum replacement medium SC^wt^ and SC^Sox2/Cl2^ reach a similar number of total live cells after three days (Supplementary Fig. 4c,d).

**Figure 3:**
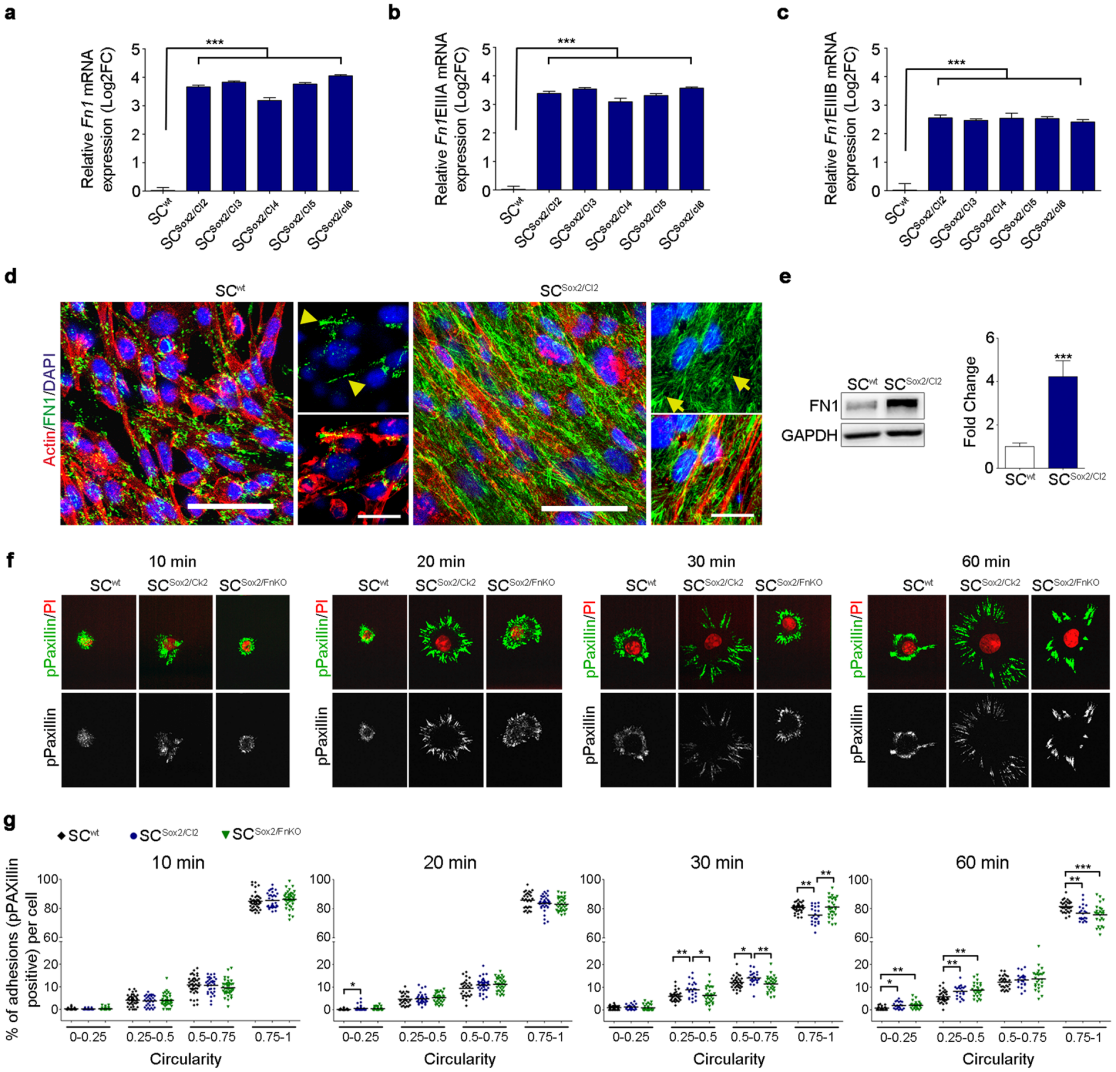
Sox2 overexpression induces FN fibrillogenesis and focal adhesion in Schwann cells. (**a**–**c**) Graphs show real-time RT-qPCR measurements of *Fn1, Fn1*EIIIA and *Fn1*EIIIB mRNA levels in SC^wt^ and selected clones after 3 days in culture. *Gapdh*, *Ankrd27* and *Rictor* were used as reference genes, values are relative expression to SC^wt^. t-test comparing each clone with the control (N = 5). (**d**) Representative confocal images of Actin fibres (red) and FN1 (green) immunostainings showing FN expression and fibrillogenesis in SC^wt^ (arrow head) an in SC^Sox2/Cl2^ (arrows) cultured in FN-free medium. Nuclei were counterstained with DAPI (blue). Scale bars, 50 μm and 25 μm. (**e**) Western blot analysis and quantification of total FN1 protein in the whole lysate of SC^wt^ and SC^Sox2/Cl2^. GAPDH was used as a loading control. (N = 8). (**f**) pPaxillin immunostaining showing the formation of focal adhesion during the first 60 minutes after seeding on laminin-coated slides. (**g**) Distribution of adhesion structures during cell spreading, according to their circularity (n ≥ 25 cells/time point). Graphs show mean value ± s.e.m, *p < 0.05, **p < 0.005, ***p < 0.0005.

We then analysed FN expression in our *in vitro* model system. 20 and 12 FN isoforms have been described in human and rodents respectively, based on the splicing combination of the three domains EIIIA, EIIIB, and V^25^. We evaluated only the isoforms present in cellular fibronectin (EIIIA and EIIIB), and found a significant up-regulation in the gene expression levels of *Fn1* and in the specific splice variants *Fn1*EIIIA and *Fn1*EIIIB upon Sox2 overexpression (Fig. 3a–c). Immunostaining and western blot analysis confirmed the upregulation of FN expression and revealed the increase in FN fibrillogenesis in Sox2-positive clones (Fig. 3d,e and Supplementary Fig. 3c; full-length blots are presented in Supplementary Fig. 3b). As we observed that all Sox2-positive clones have an increase in FN fibrillogenesis (Supplementary Fig. 3c), we performed the subsequent experiments with SC^Sox2/Cl2^ only. Furthermore, we did not observe differences between the control clone (SC^Cl0^) and wild-type cells (SC^wt^) by immunostaining. For this reason, we continued our studies using SC^wt^. Notably, in SC^Sox2/Cl2^, FN is localized at the edges and towards the cell centre (Fig. 3d, arrows), a characteristic of fibrillar adhesion, as opposed to SC^wt^, where FN is localized only at the cell edges, typical of focal complexes and focal adhesions (Fig. 3d, Head arrows).

To test the direct implication of FN expression in the Sox2-dependent Schwann cell-matrix adhesion, we knocked out FN using the CRISPR/Cas9 technology in SC^Sox2/Cl2^ (Supplementary Fig. 4a,b). We first performed the same cell proliferation assay described before. We cultured the FN KO cell line (SC^Sox/Fn1KO^) in NM and KSRM. The results show that at day one, there was no significant differences between SC^Sox/Fn1KO^ and SC^wt^ or SC^Sox2/Cl2^. At day two and three, we only found that in the NM cell proliferation is reduced in SC^Sox/Fn1KO^ compared to SC^wt^ (day 2: p = 0.0144 and day 3: p = 0.0006). No differences in cell proliferation were found between SC^Sox/Fn1KO^ and SC^wt^ or SC^Sox2/Cl2^ cultured in KSRM (Supplementary Fig. 4c,d).

To understand the role of Sox2 and FN expression in cell-matrix adhesion, we evaluated the formation of focal adhesions in SC^wt^, SC^Sox2/Cl2^, and SC^Sox2/FnKO^ during cell spreading. We used phosphoPaxillin (pPaxillin, phosphorylation site tyr-118) as a marker of focal adhesion^26^. We quantified focal adhesions, immunostained with pPaxillin, by measuring the circularity of the adhesion structure according to Horzum *et al*.^27^. Values close to 1 refer to circular structures typical of nascent adhesion or focal complexes, and values close to 0 refer to longer structures typical of focal adhesion. We found that at 20 minutes SC^Sox2/Cl2^ formed focal adhesion structures earlier than SC^wt^ and SC^Sox2/FnKO^. After 30 and 60 minutes, we observed a significant increase in the number of focal adhesions in SC^Sox2/Cl2^ compared to SC^wt^ and SC^Sox2/FnKO^ (Fig. 3f,g). In summary, our results suggest that Sox2 overexpression induces the formation of focal adhesion as well as the production of FN in Schwann cells cultured *in vitro*.

### Sox2-induced FN fibrillogenesis is essential for Schwann cell alignment and directional migration

**Figure 4:**
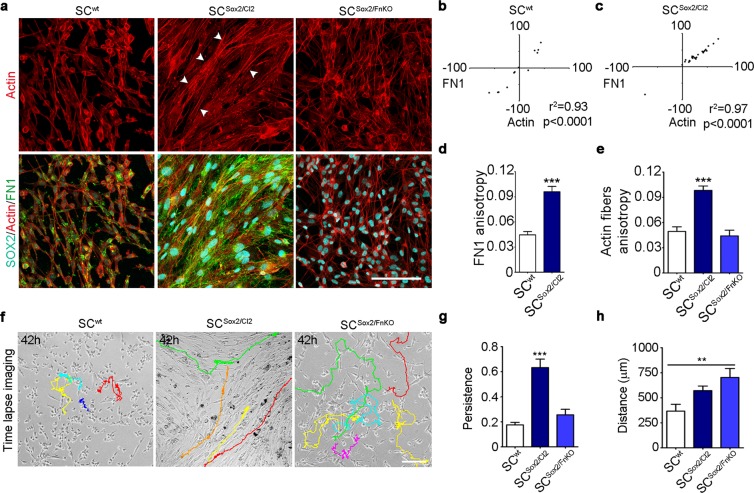
Sox2-induced FN fibrillogenesis is responsible for Schwann cell organization and persistent migration. (**a**) Representative confocal images of SOX2 (cyan), Actin fibres (red) and FN1 (green) immunostainings of SC^wt^, SC^Sox2/Cl2^ and SC^Sox2/FnKO^. Arrow heads show the organization of the actin fibres in the SC^Sox2/Cl2^. Scale bar 100 μm. (**b**,**c**) Correlation of FN1 fibre angles with the orientation of actin fibres in SC^wt^ and SC^Sox2/Cl2^ (N = 3) (**d**,**e**) Quantification of FN1 anisotropy of SC^wt^ and SC^Sox2/Cl2^ and actin fibres of SC^wt^, SC^Sox2/Cl2^ and SC^Sox2/FnKO^ (N = 3, n ≥ 12 areas) (**f**) Representative time-lapse imaging from Supplementary Movies 5–7 from SC^wt^, SC^Sox2/Cl2^ and SC^Sox2/FnKO^ respectively, cultured in KSRM (FN-free). Color lines show single-cell tracks of the Schwann cells during 42 hours. Scale bar, 100 μm. (**g**,**h**) Quantification of the distance and persistence of SC^wt^, SC^Sox2/Cl2^ and SC^Sox2/FnKO^ migration in KSR medium, quantification was performed considering only 100 frames per cell (n = 10 cells). Graphs show the mean ± s.e.m. **p < 0.005, ***p < 0.0005.

After axotomy, Schwann cells collectively migrate along the axis of the nerve stump^5^. We have shown that Sox2 promotes Schwann cell directional migration and FN fibrillogenesis. We therefore asked whether Sox2-induced FN fibrillogenesis could control Schwann cell clustering and directional migration. To test the direct implication of FN up-regulation in the Sox2-dependent Schwann cell directional migration, we evaluated anisotropy and cell behaviour of SC^wt^, SC^Sox2/Cl2^, and SC^Sox2/FnKO^. We first quantified cell alignment by analysing actin anisotropy. We found that after 3 days in culture, FN ablation in SC^Sox2/FnKO^ led to a loss of Schwann cell organization (Fig. 4a). We next analysed the direction of the actin fibres orientation, labelled with Phalloidin, and the FN fibres, stained with a FN antibody. In SC^Sox2/Cl2^, we found a significant correlation between the angles of actin and FN fibres (Fig. 4b,c) and a significant increase in cell-cell alignment (Fig. 4d,e). In contrast, we found that directionality and cell-cell alignment are lost in SC^Sox2/FnKO^ (Fig. 4e).

Previous studies have shown that migration of rat Schwann cells on laminin is dependent on β1 integrins, whereas αv integrin is important for their migration on FN ^28^. Therefore, we next assessed the gene expression level of the integrins *Itgβ1* and *Itgαv* by RT-qPCR. We found no differences in the mRNA levels of *Itgβ1* between SC^wt^ and SC^Sox2/Cl2^. However, the knockout of FN significantly decreased the gene expression levels of *Itgβ1*. *Itgαv* was significantly up-regulated in Sox2-positive Schwann cells independently of FN expression, suggesting that Sox2 overexpression modulates the expression of this integrin. Finally, we also evaluated mRNA levels of *Itgα*5 which is part of the FN receptor together with *Itgβ*1 and its expression has been previously reported in Schwann cells^28^. However, we did not find differences between Sox2-positive Schwann cells and wild-type Schwann cells at the mRNA level (Supplementary Fig. 5a–c). Therefore, we confirmed an increase in the gene expression of *Itgαv* as predicted.

To better visualize and understand cellular behaviour, we performed time-lapse imaging of SC^wt^, SC^Sox2/Cl2^, and SC^Sox2/FnKO^ over 3 days. As Supplementary Movies 5–7 show, Sox2 overexpression increases the adhesion of the cells to the substrate, and particularly the up-regulation of FN is necessary for Schwann cell directional migration. We showed that SC^wt^ or SC^Sox2/FnKO^ exhibit random movements, in contrast to SC^Sox2/Cl2^, which have a directionally persistent cell migration (Fig. 4f). Persistence and distance analysis showed that the capacity to maintain the direction of motion was lost upon FN knockout independently of the distance travelled, which was increased in this condition (Fig. 4g,h). These results demonstrate that Sox2-induced FN expression and fibrillogenesis are required for Schwann cell alignment and directional migration.

### Sox2 directly controls FN expression in Schwann cells

**Figure 5:**
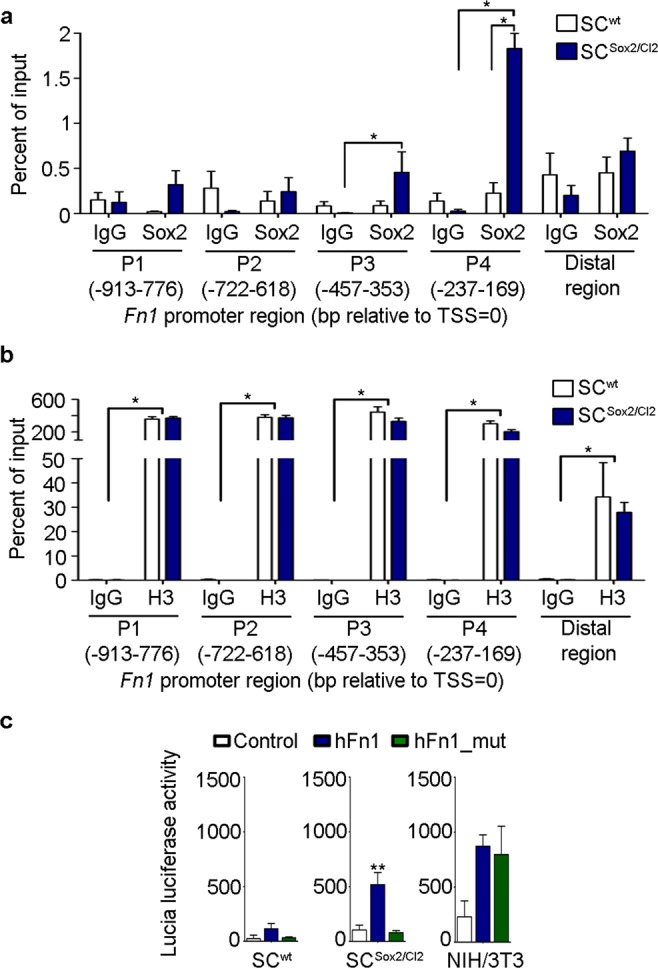
Sox2 directly controls FN expression in Schwann cells. (**a**,**b**) ChIP-qPCR assay of SOX2 binding site in the rat *Fn1* proximal promoter. H3 and IgG were used as positive and negative controls respectively. (**c**) Reporter assay showing Lucia luciferase activity 48 h post transient transfection of SC^wt^, SC^Sox2/Cl2^ (N = 5) and NIH/3T3 (N = 4) with the pDRIVE5Lucia plasmid containing the human *Fn1* promoter region (hFn1), the human *Fn1* promoter with a mutation in the Sox2 binding site (hFn1_mut) and the control plasmid without the fibronectin promoter region (control). Graphs show mean value ± s.e.m. **p < 0.005.

We then addressed the question whether FN expression in SC is directly controlled by Sox2. We performed an *in-silico* promoter analysis using the MatInspector tool. We found several potential binding regions in the rat *Fn1* promoter that can be recognized by Sox2 (Supplementary Fig. 5d,e). We also evaluated if the binding sites that we identified in rat were conserved between species. We found that the binding site located −200 nucleotides upstream of the transcription starting site (TSS) in the rat *Fn1* promoter is conserved across 8 out of 9 studied species including humans (Supplementary Fig. 5f). We therefore asked whether Sox2 controls directional collective migration of Schwann cells by direct binding to the *Fn1* promoter region, controlling its expression. We first performed chromatin immunoprecipitation assays, which confirmed that Sox2 binds to the rat *Fn1* promoter region (Fig. 4a,b), and found an enrichment of Sox2 on the binding region located −200 nucleotides upstream of the TSS. Then, we analysed the regulatory activity of Sox2 on *Fn1* in SC^wt^ and SC^Sox2/Cl2^. We used the expression of the Lucia luciferase synthetic gene as a reporter assay of the human *Fn1* (*hFn1*) promoter, which contains only one Sox2 binding site (Supplementary Fig. 5f,g). We detected low levels of Lucia luciferase activity in SC^wt^, in agreement with the low levels of FN expression detected in these cells. On the other hand, we detected significantly higher levels of Lucia luciferase activity in SC^Sox2/Cl2^ expressing the plasmid containing the *hFn1* promoter, than in the control plasmid without the FN promoter region (Fig. 5c). To demonstrate that the expression of the Lucia luciferase reporter was directly regulated by Sox2, we performed a site-directed mutagenesis of the Sox2 core-binding site in the human *Fn1* promoter (Supplementary Fig. 5g). We observed a significant reduction of the reporter expression to below basal levels in SC^Sox2/Cl2^. As a control of the assay, we used mouse NIH/3T3 fibroblasts, a cell line with high levels of FN expression but negative for Sox2. As expected, we observed a significant increase in the Lucia luciferase signal, but no differences observed after mutating the Sox2 binding site (Fig. 5c). These results confirmed that Sox2 acts as a strong transcriptional activator of FN expression in our *in vitro* system.

### Axons follow Schwann cell organization *in vitro*

**Figure 6:**
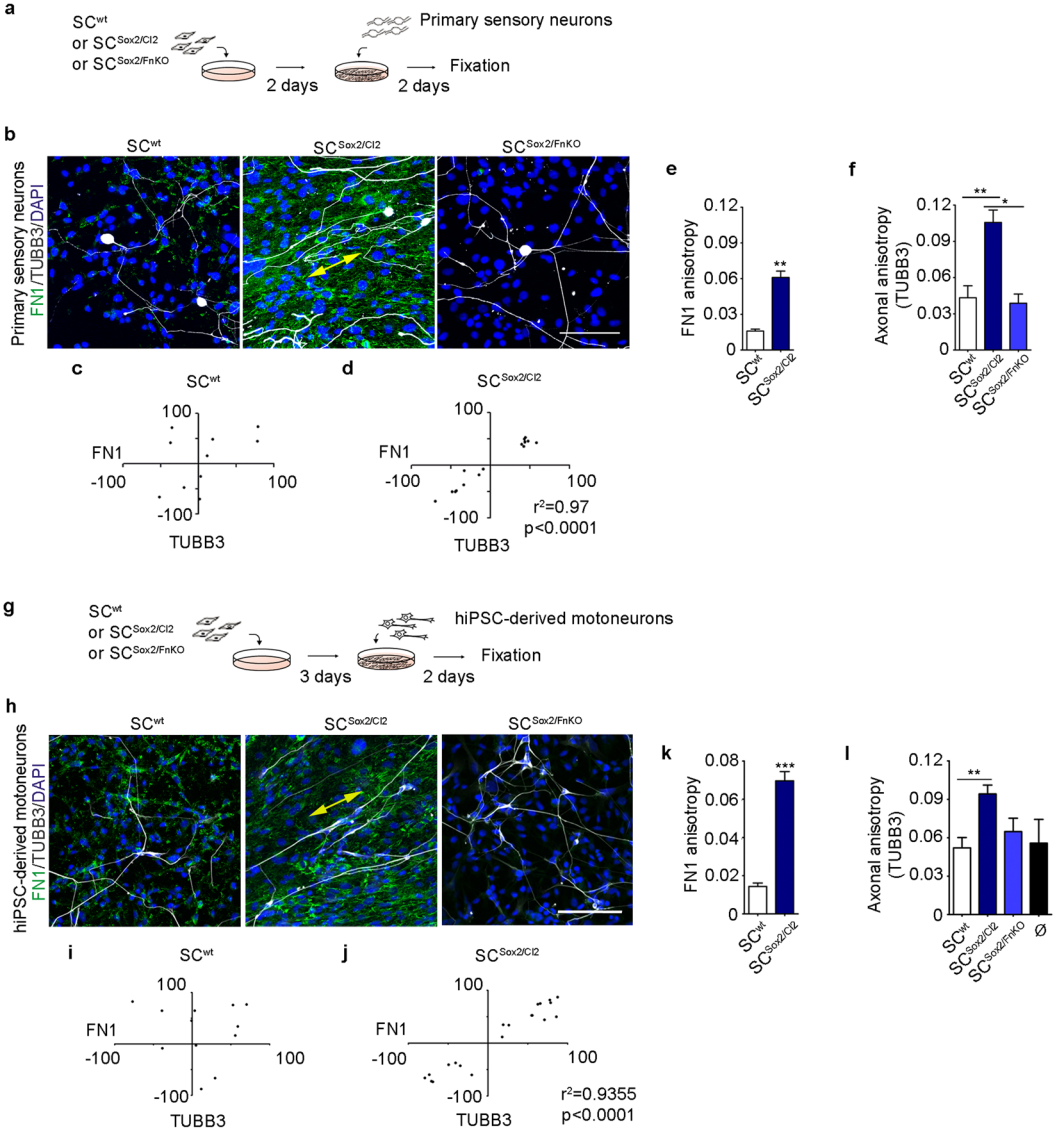
Neurons align their axons on FN-dependent organized Schwann cells. (**a**) Experimental design of Schwann cells (SC^wt^, SC^Sox2/Cl2^ or SC^Sox2/FnKO^) co-cultured with mouse primary sensory neurons. (**b**) Representative confocal images of FN1 (green) and TUBB3 (white) immunostainings of primary sensory neurons co-cultured with SC^wt^ (129 cells), SC^Sox2/Cl2^ (142 cells) or SC^Sox2/FnKO^ (120 cells). Nuclei were counterstained with DAPI (blue). Double head arrow shows the alignment of axons with FN fibres in SC^Sox2/Cl2^. Scale bar, 100 μm. (**c**,**d**) Correlation of FN1 fibre angles of SC^wt^ and SC^Sox2/Cl2^ with the axonal angles (TUBB3) (N = 3). (**e**,**f**) Quantification of FN1 fibres (N = 3, n ≥ 10 areas) and axonal anisotropy (N = 3, n ≥ 8 areas) in primary sensory neurons co-cultured with: SC^wt^, SC^Sox2/Cl2^ and SC^Sox2/FnKO^. (**g**) Experimental design of Schwann cells (SC^wt^, SC^Sox2/Cl2^ or SC^Sox2/FnKO^) co-cultured with human iPSC-derived motoneurons. (**h**) Representative confocal images of FN1 (green) and TUBB3 (white) immunostaining of human iPSC-derived motoneurons co-cultured with SC^wt^ (211 cells), SC^Sox2/Cl2^ (231 cells) or SC^Sox2/FnKO^ (245 cells). Nuclei were counterstained with DAPI (blue). Double-head arrow shows the alignment of axons with FN fibres in SC^Sox2/Cl2^. Scale bar, 100 μm. (**i**,**j**) Correlation of FN1 fibre angles of SC^wt^ and SC^Sox2/Cl2^ with the angles of human iPSC-derived motoneurons (TUBB3) (N = 3). (**k**,**l**) Quantification of FN1 fibres (N = 3, n ≥ 18 areas) and axonal anisotropy (N = 3, n ≥ 9 areas) of human iPSC-derived motoneurons co-cultured with: SC^wt^, SC^Sox2/Cl2^ and SC^Sox2/FnKO^. Ø corresponds to human iPSC-derived motoneurons cultured alone. Results are shown as the mean ± s.e.m. *p < 0.05, **p < 0.005, ***p < 0.0005.

After nerve injury, axons regrow along the axis of the nerve stumps, following Schwann cell directional migration^29^. To test whether FN-dependent Schwann cell clustering and directional migration triggered by Sox2, might be responsible for directing organized axonal growth, we cultured dissociated adult mouse dorsal root ganglion neurons (primary sensory neurons) on the different Schwann cell lines: SC^wt^, SC^Sox2/Cl2^, and SC^Sox2/FnKO^ (Fig. 6a). Interestingly, using immunostaining with antibodies that recognize FN and the pan-neuronal marker, b3-Tubulin (TUBB3), we observed that axonal processes from primary sensory neurons follow the orientation of the SC^Sox2/Cl2^ but were not oriented when plated on SC^wt^ or SC^Sox2/FnKO^ (Fig. 6b). When we analysed the angles of FN fibres and the orientation of axons using the TUBB3 staining, we found a significant correlation between FN and axons only in the presence of SC^Sox2/Cl2^ and not when primary sensory neurons were cultured with SC^wt^ (Fig. 6c,d). To confirm these observations, we analysed the anisotropy of FN fibres and axons in the different co-culture conditions. We showed that anisotropy of the axons is significantly increased in SC^Sox2/Cl2^ in comparison to SC^wt^ and that this anisotropy drops dramatically in SC^Sox2/FnKO^ (Fig. 6e,f).

Very little is known about collective migration of Schwann cells and axonal growth in humans. To explore the effect of FN-dependent Schwann cell organization on the axonal growth of human neurons, we differentiated motoneurons from human induced pluripotent stem cells (iPSC) using a standard protocol^30^ (Supplementary data). We first characterized the human derived motoneurons by immunostaining and real-time RT-qPCR (Supplementary Fig. 6a,b). We confirmed the identity of the motoneurons by the expression of the specific markers *CHAT, HB9*, and *ISL1*. We also found expression of *LHX3* and *GATA2*, markers of interneurons. We next plated these human iPSC-derived motoneurons on the three different Schwann cell cultures SC^wt^, SC^Sox2/Cl2^, and SC^Sox2/FnKO^ and proceeded with the same analysis used for the primary sensory neurons (Fig. 6g). Consistent with the data obtained with mouse primary sensory neurons, we observed orientation (Fig. 6h–j) and high anisotropy of the human motoneuron axons in the presence of SC^Sox2/Cl2^ and a loss of axonal orientation and anisotropy in SC^Sox2/FnKO^ (Fig. 6k,l). These data demonstrated that FN-dependent Schwann cell organization is important for axon guidance in human motoneurons.

### Aligned FN matrix is not sufficient to guide axonal migration

To confirm the specific role of FN in this guidance, we asked whether FN alone could promote the alignment of axons *in vitro*. To test this hypothesis, we decellularized the SC^Sox2/Cl2^ cultures before plating the dissociated primary sensory neurons (Supplementary Fig. 7a). We used a detergent-based decellularization protocol to remove the cells and the genetic material and keep the ECM in the culture dish (Supplementary Fig. 7b). We observed that following decellularization of the SC^Sox2/Cl2^ culture, FN anisotropy is maintained, but that the remaining matrix is not sufficient to support directional axonal outgrowth (Supplementary Fig. 7c–e). Analysis of FN fibres angles, from the remaining SC^Sox2/Cl2^ matrix, and axonal direction showed a complete loss of correlation between them (Supplementary Fig. 7f). Remarkably, these results indicate that direct contact between Schwann cells and axons is necessary.

We next evaluated whether Schwann cells could use exogenous FN present in the extracellular environment to help axonal guidance. To test this hypothesis, we plated SC^Sox2/FnKO^ on decellularized SC^Sox2/Cl2^ cultures where oriented FN matrix remained, as just described (Supplementary Fig. 7a). Surprisingly, we observed by immunostaining of FN and TUBB3 that axons of primary sensory neurons grew randomly in this condition, as shown in Supplementary Fig. 7c, which was confirmed by axonal anisotropy analysis, and the lack of correlation between FN fibres angles and axons (Supplementary Fig. 7e,g). We quantified SC^Sox2/FnKO^ alignment on the extracellular matrix generated by SC^Sox2/Cl2^ and found that the FN knockout cells did not align in the SC^Sox2/Cl2^ matrix (Supplementary Fig. 7h,i). These data demonstrate that FN-depleted Schwann cells are unable to use the surrounding FN matrix to get oriented and to help axonal migration. Thus in our *in vitro* model, axonal guidance occurs through direct contact with Schwann cells oriented through their own secreted FN matrix.

### FN expressed by fibroblasts does not influence axon alignment

**Figure 7:**
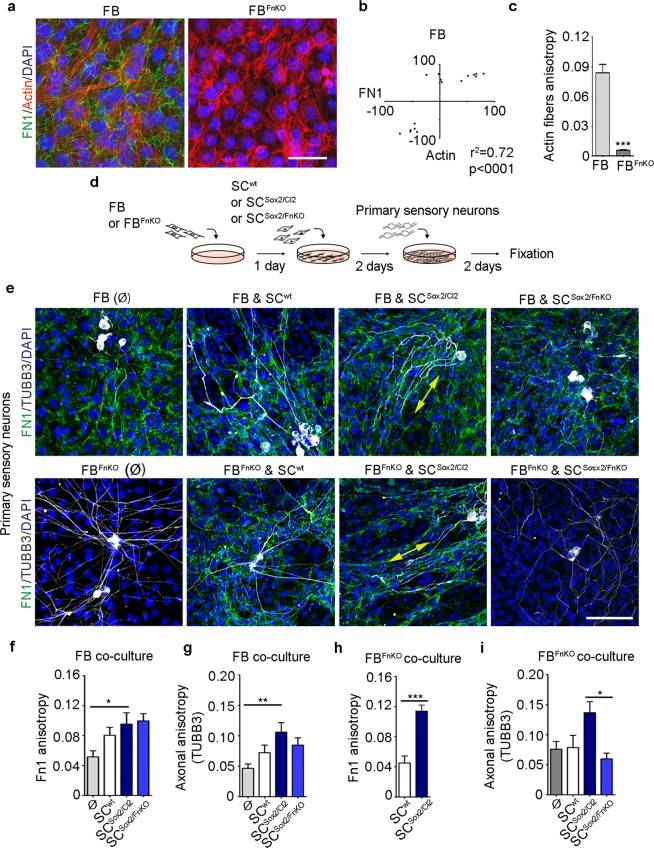
Mouse primary sensory neurons align only in the presence of Sox2-positive Schwann cells. (**a**) Representative confocal images of FN1 (green) and Actin fibres (red) immunostainings of FB and FB^FnKO^. Nuclei were counterstained with DAPI (blue). Scale bar, 100 μm. (**b**) Correlation of FN1 fibre angles with the orientation of actin fibres of FB (N = 3). (**c**) Quantification of actin fibres anisotropy of FB and FB^FnKO^ (N = 3, n ≥ 18 areas). (**d**) Experimental design of co-cultures between Fibroblasts (FB and FB^FnKO^), Schwann cells (SC^wt^, SC^Sox2/Cl2^ or SC^Sox2/FnKO^) and primary sensory neurons from adult mice. (**e**) Representative confocal images of FN1 (green) and TUBB3 (white) immunostainings of primary sensory neurons co-cultured with FB plus SC^wt^, SC^Sox2/Cl2^ or SC^Sox2/FnKO^ [top panels] and with FB^FnKO^ plus SC^wt^, SC^Sox2/Cl2^ or SC^Sox2/FnKO^ [bottom panels]. Nuclei were counterstained with DAPI (blue). Double-head arrow shows the alignment of axons with FN fibres. Scale bar, 100 μm. (**f**) Quantification of FN1 anisotropy of the primary sensory neurons and FB co-culture conditions: alone (Ø), with SC^wt^, with SC^Sox2/Cl2^ or with SC^Sox2/FnKO^ (N = 3, n ≥ 7 areas). (**g**) Axonal anisotropy (TUBB3) of the primary sensory neurons in the co-culture conditions with: FB (Ø), FB plus SC^wt^, SC^Sox2/Cl2^ or SC^Sox2/FnKO^, (N = 3, n ≥ 7 areas). (**h**) Quantification of FN1 anisotropy of the primary sensory neurons and FB^FnKO^ co-culture conditions: with SC^wt^ or with SC^Sox2/Cl2^ (N = 3, n ≥ 6 areas). (**i**) Axonal anisotropy (TUBB3) of the primary sensory neurons in the co-culture conditions with: FB^FnKO^ (Ø), FB^FnKO^ plus SC^wt^, SC^Sox2/Cl2^ or SC^Sox2/FnKO^, (N = 3, n ≥ 6 areas) Graphs show the mean value ± s.e.m. *p < 0.05, **p < 0.005, ***p < 0.0005.

We have shown that FN-depleted Schwann cells are disorganized and unable to guide themselves with the surrounding FN matrix to associate, directionally migrate, and guide axonal regrowth. Since Schwann cells are in direct contact with fibroblasts in the regenerating PNS and fibroblasts express high levels of FN, we tested the potential implication of fibroblast-secreted FN in Schwann cell clustering, migration, and axonal guidance. For this, we used a wild-type mouse fibroblast cell line (FB) and a *Fn1* knocked-out line (gifts from Prof. Reinhard Fässler). We first evaluated FN expression and the anisotropy of the FB. As expected, we observed that wild-type FB express high levels of FN, which is lost in the knocked-out cell line (Fig. 7a). The analysis of correlation between FN fibrils and actin fibres orientation in the wild-type FB line showed that the two types of fibres are correlated (Fig. 7b). Interestingly, the actin fibres of the wild-type FB presented a high anisotropy, that is lost in FB^FnKO^ (Fig. 7c). These results indicate that FN influences fibroblast orientation, confirming a general role of FN in cellular orientation.

To study axonal guidance, we evaluated eight co-culture conditions: (i) FB and primary sensory neurons; (ii) FB, SC^wt^, and primary sensory neurons; (iii) FB, SC^Sox2/Cl2^; and primary sensory neurons; (iv) FB, SC^Sox2/FnKO^, and primary sensory neurons; (v) FB^FnKO^ and primary sensory neurons; (vi) FB^FnKO^, SC^wt^, and primary sensory neurons; (vii) FB^FnKO^, SC^Sox2/Cl2^, and primary sensory neurons; and (viii) FB^FnKO^, SC^Sox2/FnKO^, and primary sensory neurons (Fig. 7d). Primary sensory neurons were oriented only when they were co-cultured on SC^Sox2/Cl2^, condition iii (Fig. 7e). Evaluation of the FN fibres and axonal anisotropy, in the eight different co-culture conditions with FB, showed a significant increase of anisotropy only in the presence of SC^Sox2/Cl2^ (Fig. 7f,g). Analysis of co-culture with FB^FnKO^ showed a similar trend, a significantly increased anisotropy of FN fibres and axons in the presence of SC^Sox2/Cl2^, condition vii (Fig. 7h,i). Interestingly, we found that SC^wt^ in the presence of FB increase their fibrillogenesis but with a lower anisotropy than SC^Sox2/Cl2^, and this fibrillogenesis was not enough to significantly influence axonal orientation. In summary, we found that axons were oriented only in the conditions where SC^Sox2/Cl2^ were present, conditions (iii) and (vii), and that FN coming from fibroblasts does not influence axonal orientation whereas FN from Sox2-expressing Schwann cells is necessary. These results confirmed that, in our *in vitro* model, axonal guidance is conducted by Schwann cells which use their own FN to orient themselves, independently of the fibroblast-secreted FN.

### FN is co-expressed with Sox2 in Schwann cells *in vivo*

To assess the significance of our findings *in vivo*, we evaluated Sox2 and FN expression after sciatic nerve transection in adult rats at days 4, 7, and 10 post-injury. We performed a complete transection of the sciatic nerve with a subsequent direct nerve coaptation, mimicking the standard clinical treatment. We used the contralateral site of the animal as a control, where we performed a similar surgical procedure to expose the nerve without transection (Supplementary Fig. 8a,b). We first evaluated GFAP and TUBB3 to confirm the regeneration process of the sciatic nerve. We observed GFAP up-regulation by immunostaining at days 4, 7, and 10 (Supplementary Fig. 8c,d). Using TUBB3, we observed the regrowth of the axons at days 7 and 10 (Supplementary Fig. 8c).

**Figure 8:**
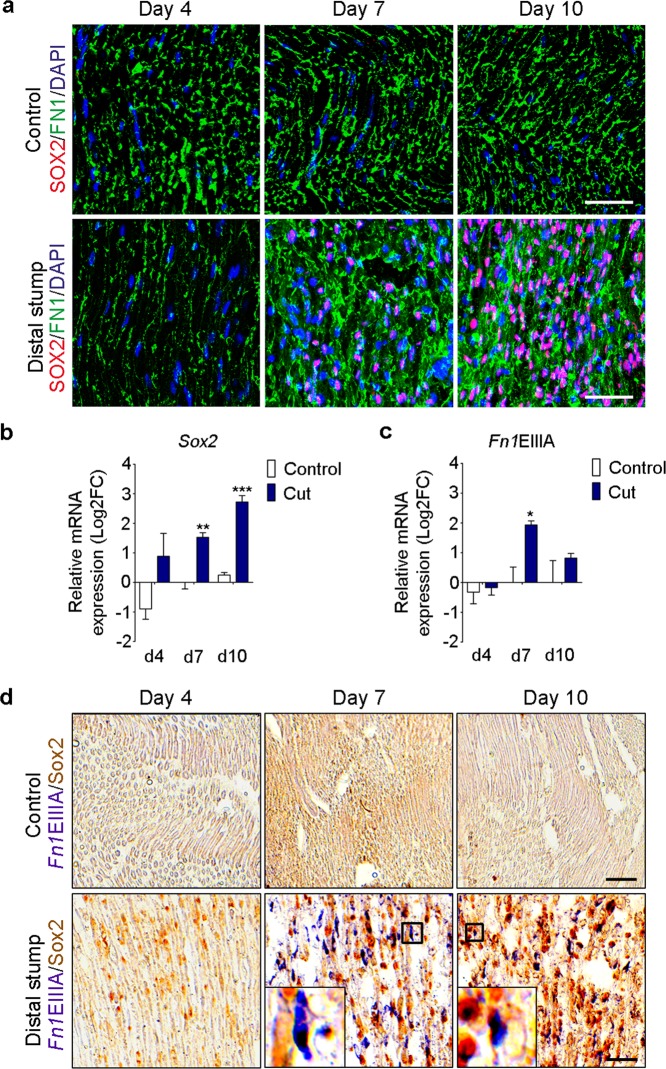
Sox2 and FNEIIIA are up-regulated and co-expressed in Schwann cells after sciatic nerve transection. (**a**) Representative confocal images of FN1 (green) and SOX2 (red) immunostainings of rat sciatic nerves in control conditions or after complete transection, at day 4, 7 and 10 post surgery. Nuclei were counterstained with DAPI (blue). Scale bar, 50 μm. (**b**,**c**) Real-time RT-qPCR measurements of *Sox2* and the *Fn1* containing the spliced domain EIIIA from control and transected nerves at day 4, 7 and 10 post surgery. *Ankrd27* and *Rictor* were used as reference genes, graphs show the mean value ± s.e.m. t-test comparing cut nerves with the controls in each time point, *p < 0.05 **p < 0.005, ***p < 0.0005. (N = 3) (**d**) *In situ* hybridization using a probe against *Fn1*EIIIA (purple) and SOX2 DAB staining (brown) in control and transected nerves at day 4, 7 and 10 post surgery. Insets in the bottom panels show the co-expression of *Fn1*EIIIA with a Sox2-positive Schwann cell at day 7 and 10 post surgery. Scale bar, 50 μm.

We detected Sox2 protein expression at day 4 post-injury in the distal stump. By day 7, Sox2-positive Schwann cells were located in the cut area and the distal stump (Fig. 8a and Supplementary Fig. 8d). Finally, at day 10, Sox2-positive cells were found in the cut region, distal, and proximal stumps (Fig. 8a and Supplementary Fig. 8d). As previously reported^6^, we found that *Sox2* mRNA expression increased over time, and we observed the highest expression at day 10 (Fig. 8b).

When we analysed FN protein expression, we observed an increase in fibril assembly at days 7 and 10, coinciding with Sox2 up-regulation (Fig. 8a). During PNS injury, the FN spliced domain EIIIA gets up-regulated and is suggested to be expressed in proregenerative Schwann cells^31,32^. When we evaluated mRNA levels of *Fn1* containing the spliced variant EIIIA (*Fn1*EIIIA), we found a peak of *Fn1*EIIIA expression at day 7 post-injury (Fig. 8c). By *in situ* hybridization, we detected the spliced domain at day 4 in the cut area, mainly around the surgery site, consistent with the immunostaining results. High *Fn1*EIIIA expression was observed in the cut area and in the distal stump at day 7. At day 10, we detected a broad expression of *Fn1*EIIIA in the proximal and distal stumps as well as in the cut area (Fig. 8d and Supplementary Fig. 8f). When we analysed the co-expression of *Fn1*EIIIA mRNA and Sox2 protein expression by DAB staining, we observed double-positive cells at days 7 and 10 (Fig. 8d). Additionally, *Fn1* expression levels were higher in the cut region independently of Sox2 expression, indicating that other cells also produce FN upon injury (Supplementary Fig. 8e). These results showed that *in vivo*, Sox2-expressing Schwann cells can produce their own FN, particularly *Fn1*EIIIA, to incorporate it in their basal lamina during regeneration of the rat sciatic nerve.

## Discussion

The ECM is a dynamic structure that mediates the interaction of different cell types to support tissue regeneration. After PNS injury, a cascade of intercellular communication between Schwann cells and the microenvironment occurs, characterized by fibroblast-mediated Schwann cell sorting, macrophages dependent-blood vessels formation that influence Schwann cell migration and axonal regrowth^6,29^. Very little is known about the molecular mechanisms by which these players modify the ECM to trigger remodelling. It is well established that this dynamic remodelling is key for proper cell movement by promoting the formation of oriented ECM fibrillary trails^33^. These trails provide forces to the surrounding sensing cells, leading to a dynamic non-random cell migration^33^. An example is the migration of neural crest cells during embryonic development, in which components of the ECM, particularly FN, present along the migratory track, are necessary for their oriented movement^34^. Here we identify Sox2-mediated FN expression by the RSC 96 Schwann cells as a major regulator of their clustering and directional migration.

As previously reported, we confirm that Sox2 expressed in Schwann cells is a major regulator of their behavioural change from repulsion to attraction. Once connected, Schwann cells communicate to create a cellular network leading to collective migration^6^. Sox2 is expressed in progenitor Schwann cells and re-expressed in proregenerative Schwann cells following nerve injury. The direct targets of Sox2 in proregenerative Schwann cells are unknown. Sox2 has been related to invasion and metastasis in several human cancers^35^. Interestingly, a study in a human ovarian cancer cell line reported that Sox2 promotes cancer cell migration, invasion and colony formation through the up-regulation of FN^36^. Here we identified a specific site in the proximal region of the FN promoter where Sox2 directly binds to induce its expression, which leads to activation of fibril formation responsible for Schwann cell clustering and oriented collective migration. Given the widespread expression of Sox2 and FN in many tissues during development, their common expression in pluripotent stem cells, and their comparable link to increased tumorigenesis, the regulation of ECM components by Sox proteins might be a general mechanism by which the cellular microenvironment is modulated to adapt to changes and promote fast tissue remodelling during homeostasis, repair, or disease development. In addition, we describe an increase in focal adhesion and FN fibrillogenesis upon Sox2 overexpression in the RSC96 Schwann cell line, which is characteristic of a classical adhesion-dependent cellular movement important for collective migration. Since this observation is based on 2D cell cultures, it would be interesting to study the dynamic of adhesion structures and movement responsible for Schwann cell migration in 3D models as well as *in vivo*.

We also demonstrate that Sox2-induced FN fibrillogenesis in the RSC96 Schwann cell line governs proper axonal growth. This process happens indirectly through the coordinated alignment of the Schwann cells. First, Schwann cells organize themselves in a FN-dependent manner, creating a FN fibril track, and then axons follow the Schwann cell orientation. It is known that Schwann cells precede regenerating axons and use the blood vessels as a migratory scaffold to guide the axons through the nerve bridge^29^. c-Jun is a key transcription factor in proregenerative Schwann cells responsible for the direct communication between them and growing axons^21^. Since Sox2 and c-Jun are co-expressed in Schwann cells, and Sox2 is not downstream of c-Jun^21^, we hypothesize that Sox2 and c-Jun play a synergistic role to first organize the Schwann cells through FN fibrillogenesis, followed by the guiding of the neurons and their survival. Studying the contribution of Sox2 and c-Jun in these processes and their role in Schwann cell/endothelial cell interaction would be of great interest for our understanding of PNS regeneration and the development of potential therapies.

During PNS regeneration, endoneurial fibroblast-like cells (EFLCs), accumulate in the injured area and participate in the scar formation. EFLCs express the neural/glial antigen 2 (NG2), which has an inhibitory effect on axon growth^37^. Morgenstern *et al*. showed that *in vivo*, NG2 positive cells localize outside the Schwann cell-axon bundles in the perineurium of uninjured nerves in rats and humans^37^. In the same study, authors showed that axons follow Schwann cells but not EFLCs *in vitro*, supporting the idea that axons are not guided by fibroblasts^37^. Furthermore, Parrinello *et al*., showed that *in vitro* EFLCs do not intermingle with Schwann cells but instead repulse them to trigger their sorting and clustering through the upregulation of Sox2^6^. Our *in vitro* results show that axons do not align when the neurons are co-cultured with mouse embryonic fibroblasts in the absence of Schwann cells, suggesting that the direct contact between neurons and Schwann cells is necessary for the alignment of the axons. Therefore, we and others demonstrate that fibroblasts do not mediate axon guidance *in vitro*.

Consistent with our results in culture, Sox2 and FN are co-expressed *in vivo* in some of the proregenerative Schwann cells upon rat sciatic nerve injury. We confirmed that FN containing the spliced domain EIIIA is highly expressed upon nerve injury in the PNS and speculate that it plays a major role in Schwann cell organization and migration for proper axonal growth, through the modulation of matrix assembly. The specific role of the FN domain EIIIA is still not well understood. This spliced domain has been suggested to affect matrix levels^38^ and to decrease cell adhesion and spreading when expressed alone in fibroblasts, leading to a rapid migration^39^. In the PNS, it is expressed upon injury in the endoneurial tubes and in Schwann cells in culture^30^, suggesting a possible role in the assembly and/or maintenance of the matrix important for the directed migration of Schwann cells. Additionally, FN isoforms containing the EIIIA and EIIIB domains are important for angiogenesis during embryonic development and wound healing^40^. Since Schwann cells and new generated blood vessels are in close contact in the injured area of regenerative nerves, it would be valuable to investigate the specific role of the FNEIIIA isoform in the dynamic between Schwann cell migration, axonal guidance, and blood vessels during PNS regeneration.

In summary, we have identified Sox2 as a key regulator of ECM remodelling during Schwann cell clustering, directional migration, and axonal guidance *in vitro*. We showed that Sox2 directly controls FN expression and fibrillogenesis in RSC96 Schwann cells to mediate their organization and axonal oriented growth. Interestingly, although fibroblasts are important players in this process, their secreted FN does not induce axonal orientation in our established *in vitro* model system. Furthermore, we confirmed FNEIIIA expression in Sox2-positive Schwann cells *in vivo* during sciatic nerve regeneration, which may have relevant implications for PNS regeneration. Further work is needed to determine whether this mechanism could be targeted in future therapeutic interventions.

## Materials and Methods

### Cell culture

The rat Schwann cell line RSC96 (American Type Culture collection, ATCC, Virginia, United States of America, Cat# CRL-2765) and the rat Schwannoma cell line RT4D6P2T (ATCC, Cat# CRL-2768) were purchased from American Type Culture Collection. The packaging cell line GP2-293 was a gift from Dr. Salvador Aznar Benitah. The mouse fibroblast cell lines Fn1flox/flox (FB) and Fibroblast Fn1KO (FB^FnKO^) were a gift from Prof. Reinhard Fässler (Max-Planck Institute of Biochemistry). They were generated by immortalization of mouse embryonic fibroblasts with the adenovirus TLarge SV-40 or with the same virus expressing “CRE-deleter”. The mouse fibroblast cell line NIH/3T3 was a gift from Prof. Magdalena Götz (Helmholtz Centre Munich). Cells were cultured in maintenance medium (High-Glucose Dulbecco’s modified Eagle’s medium, Gibco, Thermo fisher scientific, Cat# 10938-025, supplemented with 10% (v/v) Fetal Bovine Serum (FBS) and 4 mM L-Glutamine) in 5% CO_2_ and at 37 °C. For fibronectin expression studies, cells were cultured in KSR medium (Knock-out DMEM medium, Gibco, Thermo Fisher Scientific, Cat# 10829-018) which is FN free, and supplemented with 10% (v/v) Knock-out serum replacement (Gibco, Thermo Fisher Scientific, Cat# 10828-028) and 4 mM L-Glutamine. Due to the low adherence of the cells to the substrate in KSR medium, we seeded them on laminin-coated plates (5 μg/ml). For all experiments, cells were cultured at a density of 23.000 cells/cm^2^.

### Retroviral transduction

GP2-293 packaging cell were co-transfected with pMXs-Sox2 (Gift from Shinya Yamanaka, Addgene, Cat# 13367) and pCMV-VSV-G (Addgene, Cat# 8454) vectors using the Xfect reagent (Clontech takara, California, United States of America, Cat# 631318) in accordance with the manufacturer’s instructions. Briefly, 20 μg of DNA from each vector plus 12 μl of Xfect reagent (Final volume 600 μl) were added to a 10 cm dish of confluent packaging cells containing 9 ml of medium. The pMXs-empty plasmid (Cell Biolabs, Inc. California, United States of America, Cat# RTV-010) was used as a negative control for Sox2 expression. Virus-containing supernatant was collected from the 10 cm dish 48 h after co-transfection and concentrated in 300 μl of maintenance medium. RSC96 cells were seeded at a density of 2,600 cells/cm^2^. 48 h after seeding, cells were treated with medium containing the viral particles (Ratio 1:5 of virus suspension to the maintenance medium) in the presence of 10 μg/ml polybrene for 24 h. Sox2+ clones were selected using a single cell-derived culture in a 96-well plate and kept in culture as we previously described. The following primer set was used to select control clones transduced with the empty vector: Fwd: 5′-gcttggatacacgccgcccacgt-3′; Rev: 5′-cccagtcacgacgttgtaaaacg-3′; 35 cycles for semiquantitative PCR.

### Axotomy and reattachment of rat sciatic nerves

Animal studies were approved by the government of Upper Bavaria and were carried out in accordance with the approved guidelines. All surgical procedures were performed under general anaesthesia (2 mg/kg midazolam, 150 µg/kg medetomidine, and 5 µg/kg fentanyl) and aseptic conditions. In female Sprague-Dawley rats (280–310 g, from Charles River, Massachusetts, United States of America), the sciatic nerve was bilaterally exposed and then transected on one site 5 mm proximal to its branching. Following transection, the nerve was subsequently coaptated with 11/0 sutures. The non-transected contralateral site served as control. Following nerve repair or nerve exposure, the biceps femoris muscle was carefully sutured back into place, the skin incision was closed and the anaesthesia reversed (0.75 mg/kg atipamezole, 200 µg/kg flumazenile, and 150 µg/kg naloxone). For postoperative analgesia, the rats received metamizol (100 mg/kg) directly after waking up, and meloxicam (0,8 mg/kg) every 24 h as well as metamizol every 6 hours for 3 days. At day 4, 7 and 10 following surgery, three of the rats were anesthetized as described above, and the sciatic nerve was bilaterally harvested. Whole trunk samples containing the micro surgically reconstructed section and the area at the same level of the control site were fixed overnight in 4% paraformaldehyde (PFA) at 4 °C. After fixation, the nerves were kept in 30% sucrose solution for 24 hours at 4 °C. They were finally embedded in OCT medium and frozen at −80 °C. Longitudinal cryosections of 12 μm were used for immunostainings and *in situ* hybridization. After nerve harvest, the rats were euthanized by intraperitoneal lethal injection of sodium pentobarbital (320 mg per animal).

### Live-cell imaging

Schwann cells and Sox2-positive Schwann cells were seeded at a density of 23.000 cells/cm^2^. Time-lapse imaging of Schwann cells was recorded every 25 min for 85 hours at 37 °C in 5% CO_2_ using the EVOS FL Cell Imaging System (Thermo Fisher Scientific, Massachusetts, United States of America) with a 10X objective. All images were processed with FIJI software package^41^ and analysed using the MTrackJ ImageJ plug-in^42^. For quantification, a minimum of 10 cells/condition were tracked during the last 42 hours and the distance and persistence were evaluated. Persistence was calculated as the displacement divided by the total distance travelled by the cell.

### Immunocytochemistry

Cells were seeded at a density of 23.000 cells/cm^2^ and fixed after 3 days in culture with 4% PFA for 20 min and permeabilized with 0.3% Triton X-100 in 0.5% Bovine serum albumin/Phosphate buffer saline (BSA/PBS) for 30 min. Primary antibodies were incubated in 1% BSA/PBS overnight at 4 °C (F-Actin with the Phalloidin peptide 1:200, Anti-CHAT 1:200, Anti-FN1 1:1.000, Anti-GFAP 1:200, Anti-HB9 1:50, Anti-PhosphoH3 1:200, Anti-ISL1 1:200, Anti-NFH 1:400, Anti-HB9 1:50, Anti-Paxillin 1:50, Anti-pPaxillin 1:50, Anti-Sox2 (Mouse for immunocytochemistry) 1:100, Anti-Sox2 (Goat, for immunohistochemistry) 1:100, Anti-TUBB3 1:500). Secondary antibodies (dilution 1:500) and DAPI (1:100) were incubated in 1% BSA/PBS for 1 hour at room temperature. Finally, samples were mounted in microscope slides using Mowiol as a mounting medium. Antibody references are summarized in Supplementary Information, Table 1. Images were acquired using a confocal microscope (LSM 510; Carl Zeiss, Inc., Jena, Germany) and LSM software (Carl Zeiss, Inc.) and for the activation of downstream signalling of Fibronectin a Zeiss Axio Observer inverted microscope (Carl Zeiss, Inc) was used. Phase-contrast images were acquired with the EVOS FL Cell Imaging System (Thermo Fisher Scientific). Images were processed with ImageJ software package^41^. Analysis of anisotropy and fibres orientation was done using the FibrilToll ImageJ plug-in^24^. Images were captured avoiding saturated pixels, a region of interest (ROI) was defined and used to quantify the fibril orientation and the anisotropy of the fibres inside the ROI. Analysis of focal adhesion after 10, 20, 30, and 60 minutes were performed according to Horzum *et al*.^27^. Images for high content analysis of pPaxillin and Paxillin staining were analysed using the Columbus® software version 2.5.0 provided by Perkin Elmer, USA. For quantification, the image region was defined by applying the building block ‘Find Image Region’ to the GFP channel. The next building block ‘Select Population’ removed border objects. Morphological parameters were calculated using the command ‘Calculate Morphology Properties’.

### Microarray analysis

To identify Sox2 target genes and pathways with a focus on ECM, we measured expression arrays of the different Sox2-positive Schwann cells and control cells. Raw and analysed microarray data is under the GEO accession number: GSE94590.

#### Data processing

Slides were measured on an Agilent DNA Microarray Scanner (G2539A) using one colour scan setting for “Agilent SurePrint G3 Rat Gene Expression 8 × 60 K Microarray” (amadid ID: 028279). The intensity data of each individual hybridization were extracted and the quality was assessed with the Feature Extraction software 10.7 (Agilent). Measured raw intensities of 62976 probes were corrected for background noise using the normexp method. Quantile normalization was applied to assure comparability across samples. The intensities of the probes were log2 transformed before statistical evaluation. P-values were computed by using a moderated t-test with help of the “limma” R/Bioconductor package (bioconductor.org) to determine significant difference between groups of three replicates for each clone and the control. P-values were adjusted for multiple testing using the method according to Benjamin & Hochberg^43^ to derive the expected percentage of false positives (i.e. the false discovery rate (FDR)).

#### Venn diagram

A Venn diagram was constructed between probes of four clones from the microarray with the following adjustments: Significance cut-offs were set at false discovery rate (FDR) adjusted p-values < 0.002 to consider the expected percentage of false positives. Only probes with a fold change (FC) greater than 2 and lower than −2 were selected for each clone. The number of different genes in each dataset was determined by summarizing the Agilent probe identifiers to their corresponding gene symbols. To detect the number of up- and downregulated genes for each intersection of the Venn diagram the average FC of probes per gene was calculated.

#### Heat map

The expression profile of normalized log2 transformed raw data from the overlap of four clones determined by the Venn diagram as well as from control cells was represented by a heat map. For the generation of the heat map we used the heatmap.2 function within the gplots package of R statistical software (www.r-project.org). Agglomerative hierarchical clustering by the hclust function (method = “complete”) was applied to group experimental conditions (columns) as well as probe intensities of genes (rows) for the heat map. Rows were scaled and represented as z-score. A dendrogram was shown for columns as well as rows. To assign subtrees of the row dendrogram, representing similar gene expression profiles as a result of the hierarchical clustering procedure to biological functions, the tree was cut in order that eight cluster remain. All genes of each cluster were then investigated separately for enrichment in the Gene Ontology (GO) category “biological process” and “cellular component” with help of the Pathway Studio program, version 11.4 (Elsevier). The top significantly up- and downregulated genes across four clones, which are assigned to GO terms “extracellular space”, “extracellular region”, “proteinaceous extracellular matrix” or “extracellular matrix” were highlighted next to the heat map.

#### Pathway analysis

Significantly expressed genes from the overlap by the Venn diagram of four clones were ranked according to their fold change (FC) and assigned to the Gene Ontology (GO) categories extra cellular matrix (ECM) and cell-adhesion. From the top ranked list genes were selected for experimental validation. Further, the genes from the overlap of the Venn diagram were explored for enrichment in Gene Ontology (GO) categories “biological process” and “cellular component” by the Pathway Studio software (version 11.4) using Fisher’s exact test and subsequent P-value correction for multiple testing by Benjamini & Hochberg^43^. Genes from the GO categories “cell adhesion” and “cell migration” were further analysed with help of the Network Builder of the Pathway Studio software (Elsevier) to generate a literature-derived network based on text mining for direct interactions between the input genes.

### *In silico* promoter analysis

The MatInspector (Genomatix, Munich/Germany) program^44^ was used to identify potential Sox2 binding sites (BSs) of the V$SORY family within the promoter sequences of Fn1. For the detection of V$SOX2.01 and V$SOX3.01 BSs within the FN1 promoter sequences the optimized matrix similarity was used.

#### Rat fibronectin1

The promoter sequence of rat fibronectin 1 (Fn1) was derived from the retrieval database ElDorado (Genomatix), version 12-2013 (NCBI build 5). Genomatix/Entrez Gene identifier: GXP_241420/25661. Position weight matrices were applied according to Matrix Family Library Version 9.1.

#### Human fibronectin 1

The promoter sequence of human fibronectin 1 (FN1) was derived from the retrieval database ElDorado (Genomatix), version 02-2016 (GRCh38). Genomatix/Entrez Gene identifier: GXP_106290/2335. Position weight matrices were applied according to Matrix Family Library Version 9.4.

#### Evolutionary conserved Sox2 binding sites

Promoter sequences of fibronectin 1 from nine different species were aligned with the DiAlign TF program in the Genomatix software suite GEMS Launcher to evaluate overall promoter similarity and to identify conserved Sox2 binding sites. The corresponding position weight matrix V$SOX2.01 was applied for detection of potential binding sites according to the Matrix Family Library, version 9.3 (March 2015). The promoter sequences were defined as in ElDorado (version 02-2016). The Genomatix/Entrez Gene identifier were used for FN1: GXP_106290/2335 (human); GXP_1045519/613269 (rhesus monkey); GXP_5899143/459926 (chimp); GXP_223655/14268 (mouse); GXP_241420/25661 (rat); GXP_1651307/100034189 (horse); GXP_3882392/280794 (cow); GXP_3541955/397620 (pig); GXP_951696/100014118 (opossum). Binding sites were considered as conserved when the promoter sequences could be aligned in the region of the Sox2 binding site with help of the DiAlign TF program by using optimized matrix similarity.

### Statistical analysis

The statistical analysis was performed with GraphPad Prism version 5.00 for Windows (GraphPad Software, San Diego California USA). Quantifications of anisotropy, persistence, distance, RT-qPCR, and FN1 western blot, were analysed with a parametric Student’s t-test (comparison of two experimental groups) or a one-way ANOVA (comparison of three or more experimental groups) with a post hoc Tukey’s test. Western blots (Sox2, pPaxillin, and Paxillin) and High content-imaging analysis were performed using a non-parametric Mann-Whitney test (comparison of two experimental groups). Correlations were done computing the Pearson correlation coefficients. Animal sample size calculation for *in vivo* sciatic nerve surgery was performed using a t-Test for Pearson Correlation. Experiments were repeated at least 3 times (N = number of independent experiments, n = number of data points) and data are presented as a mean ± s.e.m, differences with p < 0.05 were considered significant. The proliferation assay was analysed using a two-ways ANOVA for each day considering the factors cell line and medium followed by the Tukey’s multiple comparison test to evaluate differences between the cell lines cultured in DMEM or KSR medium and a Bonferroni’s multiple comparisons test to evaluate the differences between the same cell line cultured in the two different media.

## Supplementary information

Supplementary information.

Supplementary dataset.

Supplementary movie 1.

Supplementary movie 2.

Supplementary movie 3.

Supplementary movie 4.

Supplementary movie 5.

Supplementary movie 6.

Supplementary movie 7.
