# Supplementary material for: Sox2 controls Schwann cell self-organization through fibronectin fibrillogenesis
Source: Sci Rep. 2020 Feb 6;10:1984. doi: 10.1038/s41598-019-56877-y (PMC7005302; doi:10.1038/s41598-019-56877-y)
Supplement: Supplementary file 1 — Supplementary information. [file 41598_2019_56877_MOESM1_ESM.docx]

**Supplemental Information**

**Sox2 controls Schwann cell self-organization through fibronectin fibrillogenesis**

Elen Torres-Mejía^1,2,3,9^, Dietrich Trümbach^3^, Charlotte Kleeberger^8^, Ulf Dornseifer^8^, Tanja Orschmann^1,3,5^, Theresa Baecker^1^, Jara Kerstin Brenke^4^, Kamyar Hadian^4^, Wolfgang Wurst^3,6,7^, Hernán López-Schier^2^ and Sabrina C. Desbordes^1,3,5*^

^1^Stem Cells in Neural Development and Disease group. ^2^Research Unit Sensory Biology and Organogenesis.  ^3^Institute of Developmental Genetics. ^4^Assay Development and Screening Platform. ^5^Stem Cell Based-Assay Development Platform. Helmholtz Zentrum München, German Research Center for Environmental Health. 85764 Munich-Neuherberg, Germany.

^6^Chair of Developmental Genetics, Technische Universität München-Weihenstephan, 85350 Freising-Weihenstephan, Germany.

^7^German Center for Neurodegenerative Diseases (DZNE), 81377 Munich, Germany.

^8^Department of Plastic, Reconstructive, Hand and Burn Surgery, Academic Hospital Bogenhausen, Munich 81925, Germany.

*Corresponding author: Dr Sabrina C. Desbordes

**Supplemental information**

**Supplementary methodology**

**Immunohistochemistry.** Sections were washed with PBS and incubated in Buffer citrate (10 mM sodium citrate and 0.05% Tween-20, pH: 6.0) for 8 minutes at 200W in a microwave. Samples were permeabilized for 45 minutes at room temperature with 0.2% Triton X-100 and 1% BSA in PBS. Primary and secondary antibodies were incubated following the same protocol explained in the immunocytochemistry section.

***In situ* hybridization of fresh frozen sciatic nerve sections.** Fibronectin EIIIA fragment was first cloned from a pGEM-2 vector containing the fibronectin spliced domain EIIIA (gift from Dr. Richard Hynes, Addgene plasmid #14059) into the pCS2+ plasmid using the restriction enzyme EcoRI for probes generation. Sense and Antisense cRNA probes for the rat Fibronectin EIIIA fragment were generated from the newly made plasmid using the DIG RNA labelling Kit (Roche Diagnostics, Basel, Switzerland, Cat# 11175025910). Probes were purified using Illustra microspin G-50 columns (GE Healthcare, Little Chalfont, United Kingdom, Cat# 27-5330-01) and diluted 1:100 in the hybridization buffer. Sections were acetylated for 10 minutes and incubated for 2 hours with the hybridization buffer. Probes were denatured at 95ºC for 10 minutes and incubated with the sections overnight at 70ºC in a moist chamber. Slides were washed with 0.2X SSC solution at 70ºC (2x 30 minutes), 0.2X SSC at room temperature for 5 minutes and with the buffer 1 (0.1M Tris-HCl pH:7.5 and 0.15 M NaCl) for 5 minutes. Samples were blocked for 2 hours with the blocking solution (10% donkey serum in buffer 1) and incubated overnight with Anti-digoxygenin (1:2500) in the blocking solution at 4ºC. Sections were washed 3x5 minutes with Buffer 1 at room temperature and 2x 10 minutes with NTMT buffer. Sections were incubated with NBT (ROCHE, dilution, 1:286) and BCIP (ROCHE, dilution, 1:286) in 1X NTMT and 5% PVA, at 37ºC until the signal was visible. Slides were washed 3 x 10 minutes in PBS to stop the reaction and then were used for DAB staining.

**DAB staining.** After *in situ* hybridization, sections were processed for DAB staining. The tissue was permeabilized with 0.2% Triton X-100 in 10% Donkey serum/Phosphate buffer saline (Blocking solution) for 45 min. Then, samples were incubated with the primary antibody (Sox2) in the blocking solution overnight at 4°C in a humidified chamber. Sections were incubated with the biotinylated antibody diluted in the blocking solution (1:230) for 1 hour at room temperature. DAB staining was done according to the manufacturer’s protocol (Vector Kit, Vector Labs, California, United States of America, Cat# VEC-PK-6101). Sections were washed with milliQ water and mounted using the aquatex mounting medium. Images were collected with the Mirax Desk scanner v1.9 (Carl Zeiss) and processed with the software Panoramic Viewer version 1.15.2 (3DHISTECH, Budapest, Hungary).

**Immunoblotting.** Cells were collected after 3 days of culture and lysed in cold Radio-Immunoprecipitation Assay (RIPA) buffer supplemented with protease inhibitors (ROCHE, 11873580001) and 5 mM EDTA. Samples were centrifuged for 45 min at 16,000 rcf at 4°C. Protein concentration was measured with the BCA protein kit (Thermo Scientific, Cat# 23227). 10ug of total protein were loaded and separated in 8% sodium dodecyl sulphate–polyacrylamide gel and transferred to a PVDF membrane (Roche, 3010040001). Transfer was performed with a wet system (BioRad, California, United States of America,) during 14 hours, at 90 mA and at 4°C. Membranes were blocked for 1 hour with 5% milk in 50 mM Tris buffer, 150 mM NaCl and 0.1% Tween (TBS-T buffer) and incubated with the primary antibodies overnight in blocking solution (5% milk or BSA)/TBS-T (Anti-GAPDH-HRP 1:50000, Anti-FN1 1:4000, Anti-Paxillin 1:40000, Anti-pPaxillin 1:1000, Anti-Sox2 (Mouse) 1:3.000, Anti-TUBB 1:4000). Secondary antibodies (Anti-Rabbit-HRP 1:5000, Anti-Mouse-HRP 1:10000) were incubated for 1 hour at 4°C in blocking solution/TBS-T and developed using the Amersham ECL prime western blotting detection reagent (GE Healthcare Life Sciences, Little Chalfont, United Kingdom, Cat# RPN2236). Details about the antibodies are summarized in Table 1. Blots were scanned using the imaging system FUSION-SL4 advanced (Vilver Lourmat, Eberhardzell, Germany), images were analysed with the FusionCapt Advance software (Vilver Lourmat). Detection of GFAP was done using fluorescently labeled secondaries antibodies, blot was imaged using the Odyssey CLx Imaging System (LI-COR Biotechnology).

**RNA isolation, reverse transcription, semiquantitative PCR and real-time RT-qPCR.** Total RNA purification was done using the RNeasy mini kit (Qiagen, Venlo, Netherlands, Cat# 74104) and the reverse transcription was performed using the SuperScript III (Invitrogen, Thermo Fischer Scientific, Cat# 18080400). 1 μg and 20 ng of total RNA was used for the cDNA synthesis from cells and rat nerves respectively. Semiquantitative PCR was performed using the Taq PCR core kit (Qiagen, Cat# 201223) according to manufacturer’s protocol and using 0.3 μg of cDNA. Real-time RT-qPCR was performed using the qPCR master mix Sybr green (Life technologies, Thermo Fisher Scientific, Cat# [4367659](https://www.thermofisher.com/order/catalog/product/4367659)) in a 10 μl reaction containing a total of 2 ng of cDNA for *in vivo* studies or 14 ng of cDNA for *in vitro* studies and 300 nM of each primer. Real-time RT-qPCR was carried out using the QuantStudio 12K Flex (Applied Biosystems, Thermo Fisher Scientific). The list of primers and cycle conditions are summarized in Table 2. Data analysis was performed by the comparative Ct method using the Ct values and the PCR efficiencies obtained from LinRegPCR software. The control mean was calculated and all individual values (control and samples) were normalized to this mean. Fold changes were Log2 transformed and these values were used for the statistical analysis.

**Generation of fibronectin-knockout Schwann cell line.** The CRISPR/Cas9 genome-editing system was used to generate a Fn1 knockout in the Sox2-positive Schwann cells. The sgRNA sequence was designed against an upstream region of the rat *Fn1* gene (between exon 13 and 14) using the online CRISP Design Tool (http://www.e-crisp.org)^1^:

Top: CACCgAATGGAGGATAGGCTTCTCG,

Bottom: AAACCGAGAAGCCTATCCTCCATTc.

The sgRNA was cloned into the pSpCas9(BB)-2A-GFP (PX458) vector (Gift from Feng Zhang, Addgene #48138). Sox2-positive Schwann cells were transiently transfected using the Xfect transfection reagent according to the manufacturer´s protocol. GFP-positive cells were sorted using a FACSAria III (BD Biosciences, California, United States of America) and cultured for one week. A single cell derived culture was performed to select knockout clones which were confirmed by sequencing (GATC biotech, Konstanz, Germany). For subcloning of mutant alleles, PCR reactions were carried out using the Fusion High-Fidelity DNA Polymerase (BioLabs, Cat# M0530S) in a total volume of 25 μl. PCR products were subcloned using the TOPO TA cloning kit (Invitrogen, Cat# 451641) following the manufacturer’s protocol. Positive *E.coli* colonies were analysed by sequencing. Primer for amplification of the CRISPR/Cas9 targeted sequence: Fwd: 5´-TCATTTAACTGTTTCTCCACCT-3´

**Preparation of the extracellular matrix layer.** The ECM layer was generated according to Prewitz et al., ^2^. Briefly, cells were cultured at 23.000 cells/cm^2^ in laminin-coated dishes; after 72 hours, the decellularization was performed using 0.5% Triton X-100 solution in PBS supplemented with 20 mM ammonium hydroxide for 5 min. The ECM layer was washed 3 times with PBS and kept in sterile conditions for further experiments.

**Dorsal root ganglion neurons extraction and co-culture.** All animal procedures were performed according to the German Federal guidelines and approved by the Helmholtz Zentrum München Institutional Animal Care Committee. Dissociated dorsal root ganglion neurons (primary sensory neurons) were extracted from adult male CD-1 mice (Charles River) according to the JOVE protocol from de Luca et al,^3^ and cultured in laminin-coated plates (5 μg/cm^2^) at a density of 100 cell/cm^2^ in primary sensory neurons neuron medium (Neurobasal medium supplemented with: N2, B27, 2x penicillin/streptomycin, 4 mM L-Glutamine and 50 ng/ml of mouse nerve growth factor (R&D, Vienna, Austria, Cat# 1156-NG-100) for 48 hours, in 5% CO_2_ and at 37°C. For co-culture conditions, Schwann cells and fibroblasts were first seeded at a density of 23.000 cells/cm^2^ in Laminin-coated plates and cultured with KSR medium. After 48 hours, primary sensory neurons were seeded on top of Schwann cells or Fibroblasts and cultured in primary sensory neuron medium for 48 hours. For the specific co-culture condition of primary sensory neurons/Schwann cells/Fibroblasts, Fibroblasts were first seeded at a density of 11.500 cells/cm^2^ in KSR medium, after 24 hours, Schwann cells were added at the same density and after 48 hours primary sensory neurons (in primary sensory neuron medium) were seeded on top of the Schwann cells/Fibroblasts and fixed after 48 hours.

**Human pluripotent stem cell derived-motor neurons (hMN)** Fibroblast-derived iPS cells were obtained from Dr. Micha Drukker. Briefly skin fibroblasts from ATCC were reprogrammed according to Diecke et al, 2015^4^. The protocol used for the motor neuron differentiation was a modification of the one published by Qu et al^5^. Briefly, iPS cells were seeded in Geltrex-coated dishes (Life Technologies, Cat# A1413302) and maintained in mTeSR1 medium (StemCell Technologies, Vancouver, Canada, Cat# 05850), in 5% CO_2_ at 37°C. For neural induction, cells were seeded in Geltrex-coated plates at a density of 7x10^4^cells/cm^2^. Neural induction was started after 3 to 4 days when the cells reached 60-70% confluency using neural induction medium (1:1 mixture of N2 medium and B-27 medium supplemented with 100 nM LDN (ENZO, New York, USA, Cat# BV-1995-5) and 10 μM SB431542 (Miltenyi Biotech; StemMACS, Bergisch Gladbach, Germany, Cat# 130-106-275). The medium was changed every day. At day 4 and 5, 100 nM retinoic acid (Sigma-Aldrich, Missouri, United States of America, Cat# R2625) was added to the induction medium. From day 6 to day 12, 100ng/ml of human SHH C24II (Miltenyi Biotech, Cat# 130-095-727) and 100 nM of retinoic acid were added to the neural induction medium. At day 13, cells were re-seeded in polyornithine (15μg/ml, Sigma, Cat# P3655), laminin (5μg/ml, Sigma, L2020), fibronectin (500μg/ml, Sigma, Cat# F0895) and collagen I (10μg/cm2, Sigma, Cat# C0130)-coated dishes. For passaging, cells were collected with the StemPro EZPassageTM (Disposable Stem cell passaging tool, Thermo Fisher Scientific, cat#: 23181-010) in a 15 ml falcon with 2 ml medium and seeded in a ratio of 1:2 (cells:medium). The medium was supplemented with 10 μM ROCK inhibitor (Abcam, Cambridge, United Kingdom, Cat# ab120129) during the first 24 hours after re-seeding. From day 13 till 20, the medium was changed from neural induction medium to maintenance medium (1:1 mixture of N2 medium and B-27 medium supplemented with 20ng/ml human BDNF (Miltenyi Biotech, Cat# 130-096-286), 20ng/ml human GDNF (Miltenyi Biotech, Cat# 130-098-449), 0.1μM Db cAMP (Sigma, Cat# D0260), 100ng/ml IGF-1 (Miltenyi Biotech, Cat# 130-093-886), 100 ng/ml SHH and 100nM retinoic acid). The medium was changed every day. At day 21, rosettes were picked and dissociated for co-culture experiments.

**hiPSC-derived motoneurons co-culture.** Co-cultures of hiPSC-derived motoneurons and rat Schwann cells were performed under the same conditions previously described for the primary sensory neurons, using the maintenance medium supplemented with ROCK inhibitor only during the first 24 hours.

**Chromatin immunoprecipitation.** Cells were collected at day 3 and crosslinked with 1% PFA for 10 min. Chromatin immunoprecipitation was done using the MAGnify Chromatin Immunoprecipitation System (Life technology, Thermo Fisher Scientific, Cat# 492024) following the manufacturer´s protocol, using 200,000 cells/Immunoprecipitation. The Bioruptor Pico (Diagenode, Seraing, Belgium, Cat# B01060001) was used for shearing the chromatin. The conditions used were 8 cycles of 30 seconds ON, 30 seconds OFF. Immunoprecipitation was performed overnight (Anti-SOX2 (Rabbit) 1:25, Anti-H3 1:15, Anti-Rabbit IgG 1:100). Primers were designed according to the rat promoter sequence obtained from the promoter sequence retrieval database ElDorado 12-2013 (Genomatix, Munich/Germany) in the region of predicted SOX2.01 (V$SORY) binding sites. Antibody references and primers are summarized in Table 1 and 2 respectively.

**Lucia Luciferase reporter assay.** The pDRIVE-Fn1 Lucia plasmid (InvivoGen, California, United States of America, Cat# pdrive5lc-hfn) was used as a reporter assay to test the activity of Sox2 in the human Fn1 promoter. Sox2 binding site was identified by *in silico* analysis in the human fibronectin promoter and was mutated in the pDRIVE-Fn1 Lucia plasmid using the Q5 Site-Directed Mutagenesis kit (New England Biolabs, NEB, Massachusetts, United States of America, Cat# E0554) following the kit protocol. Primers were designed for a substitution of 8 nucleotides including the core sequence within the binding site using the NEBaseChanger online tool (nebasechanger.neb.com). An empty vector with the human Fibronectin promoter was generated by direct mutagenesis using the same kit; primers were design for a substitution of the entire human promoter with 3 restriction enzyme sequences (5´ XhoI SacI HindIII). Primers for generating pDRIVE-Lucia control: Fwd:5´-ctcaagcttATGGAAATCAAGGTGCTGTTTGC-3´;Rev:5´-ctcctcgagCTAGTGGGCCC

TGCAGGA-3´. Primers for generating pDRIVE-Fn1Lucia Sox2 binding site mutation: Fwd:5´-cccgGCTGCGAACCCACAGTCC-3´;Rev:5´- ggggGAGATGCTGATGGCCC

GC-3´. Cells were seeded in a 48 well/plate coated with laminin at a density of 23.000 cells/cm2 with KSR medium. Cells were transiently transfected after 48 hours, with the Xfect according to the manufacturer´s conditions, using 0.5μg of DNA. The NIH/3T3 cell line was used as a control of Fibronectin expression. These cells were cultured in the same conditions as described for Schwann cells but the transfection was performed with the TransIT-S2 Dynamic Delivery System (Mirus Bio, Wisconsin, United States of America, Cat# MIR 6003) following the manufacturer´s protocol with a ratio of 1:3 (DNA to reagent). Lucia Luciferase expression was measured 72 hours post transfection with the Quantic-Luc reagent (InvivoGen, Cat# rep-qlc1) according to the manufacturer´s protocol. Measurement conditions: In an opaque 96-well plate, 20μl of the cell medium mixed with 100μl of the Quantic-Luc reagent, 4 seconds of incubation and 0.1 reading time.

**Proliferation assay.** 80.500 cells (density: 23.000 cells/cm^2^) were seeded on laminin-coated 12 well/plate and cultured in maintenance medium (DMEM medium, High-Glucose Dulbecco's modified Eagle's medium, Gibco, Thermo fisher scientific, Cat#10938-025, supplemented with 10% (v/v) Fetal Bovine Serum (FBS) and 4 mM L-Glutamine) or in KSR medium (Knock-out DMEM medium, Gibco, Thermo Fisher Scientific, Cat#10829-018) and supplemented with 10% (v/v) Knock-out serum replacement (Gibco, Thermo Fisher Scientific, Cat# 10828-028) and 4 mM L-Glutamine in in 5% CO2 and at 37°C. Total number of live cells were counted every 24 hours during three days using trypan blue.


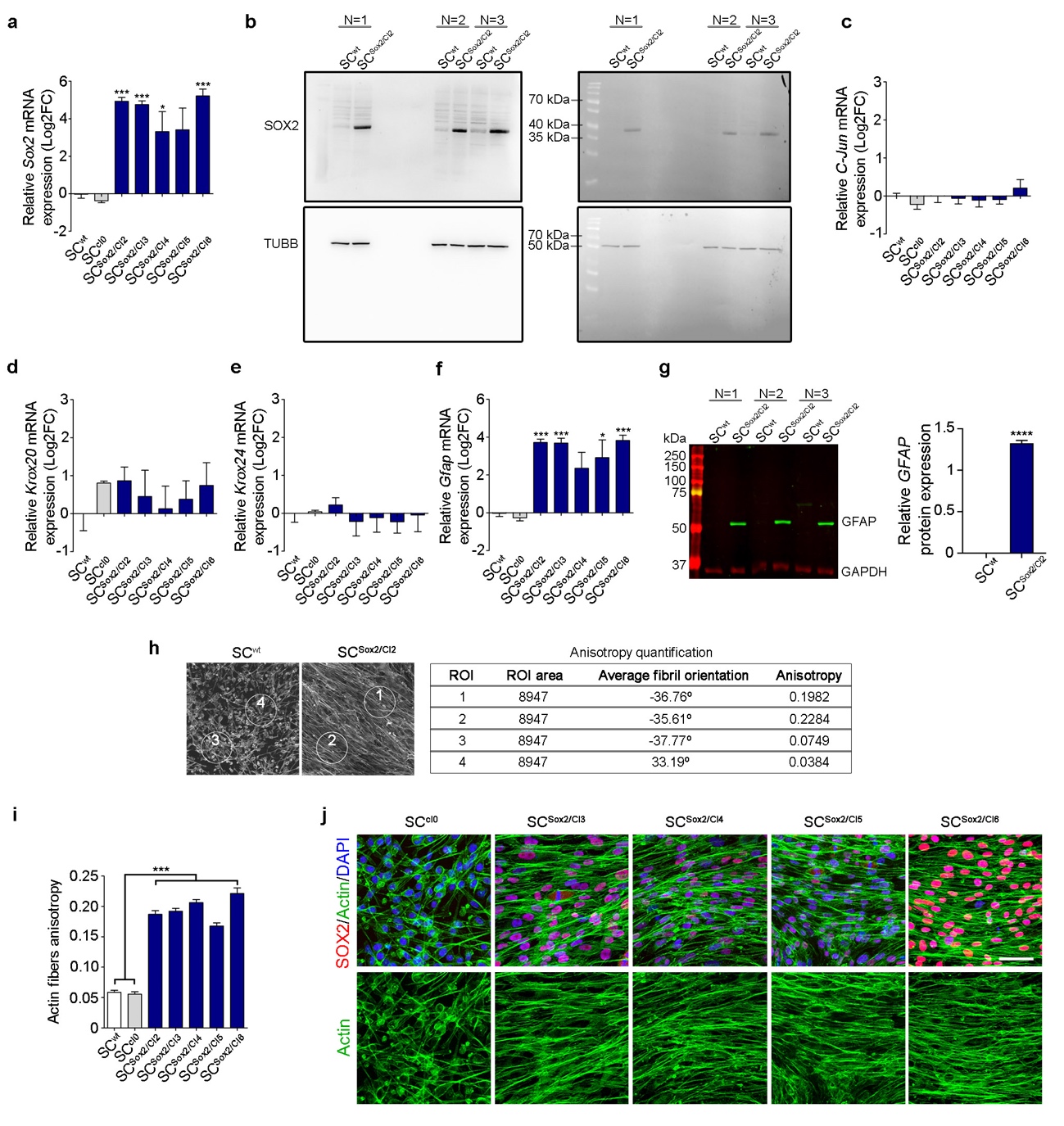


**Supplementary figure 1. Sox2 expression changes Schwann cell behaviour.** (a) Real-time RT-qPCR of mRNA levels of *Sox2*. Gene expression was normalized relative to *Gapdh, Ankrd27* and *Rictor* (N=4), t-test comparing each clone with the control. (b) Western Blot analysis of total SOX2 protein levels in the whole lysate of SC^wt^ and SC^Sox2/Cl2^. TUBB was used as a loading control (N=3), left panels were used for the quantification showed in Fig.1c, and right panels show the molecular weight of the protein of interest. (c-f) Real-time RT-qPCR of mRNA levels of different Schwann cell markers. Gene expression was normalized relative to *Gapdh, Ankrd27* and *Rictor* (N=4), t-test comparing each clone with the control. (g) Western blot image and quantification of GFAP protein levels in the whole lysate of SC^wt^ and SC^Sox2/Cl2^ normalized to GAPDH as loading control (N=3). (h) Example of anisotropy quantification using the ImageJ plug-in FibrilTool^8^; images were captured avoiding saturated pixels, and regions of interest (ROI) with the same area were selected to quantify the fibril orientation and the anisotropy of the fibres inside the ROI. (i) Quantification of actin fibre anisotropy by actin staining of the different SC (N=3). (j) Representative immunostaining confocal images of SOX2 (red) and Actin fibres (green) of SC^wt^ and selected clones in FBS-supplemented medium. Nuclei were counterstained with DAPI (blue). Scale bar, 100 μm. Graphs show mean value ± s.e.m, *p<0.05, ***p<0.0005, ****p<0.00005.


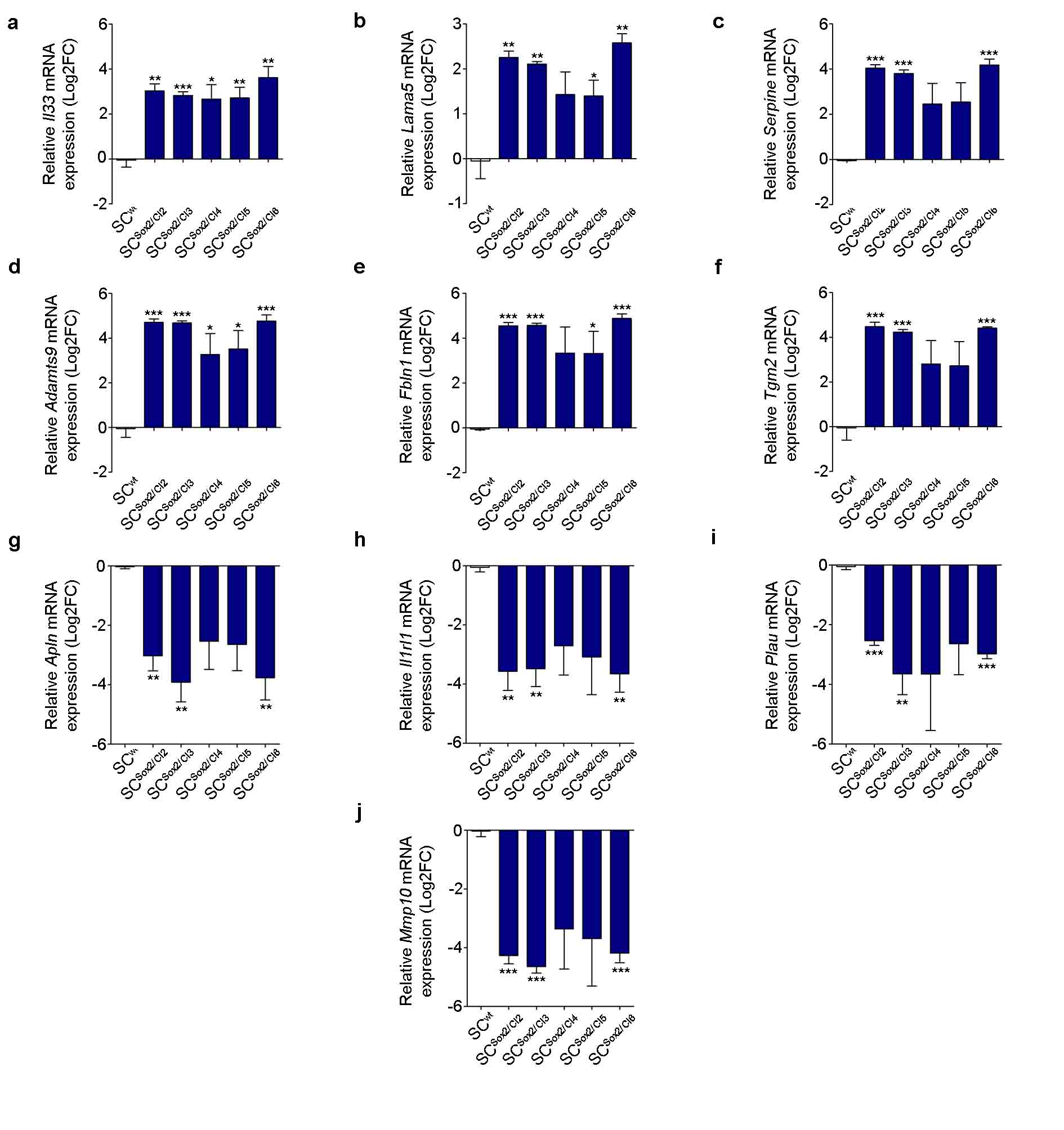
**Supplementary figure 2.** **Validation of extracellular matrix- and cell adhesion-related top genes differentially regulated in the microarray assay.** (a-j) Graphs show real-time RT-qPCR of mRNA expression in the SC^wt^ and in the selected clones. Gene expression was normalized relative to *Gapdh, Ankrd27* and *Rictor*. t-test comparing each clone with the control. Results are shown as the mean ± s.e.m (N=4). *p<0.05, **p<0.005, ***p<0.0005.

**
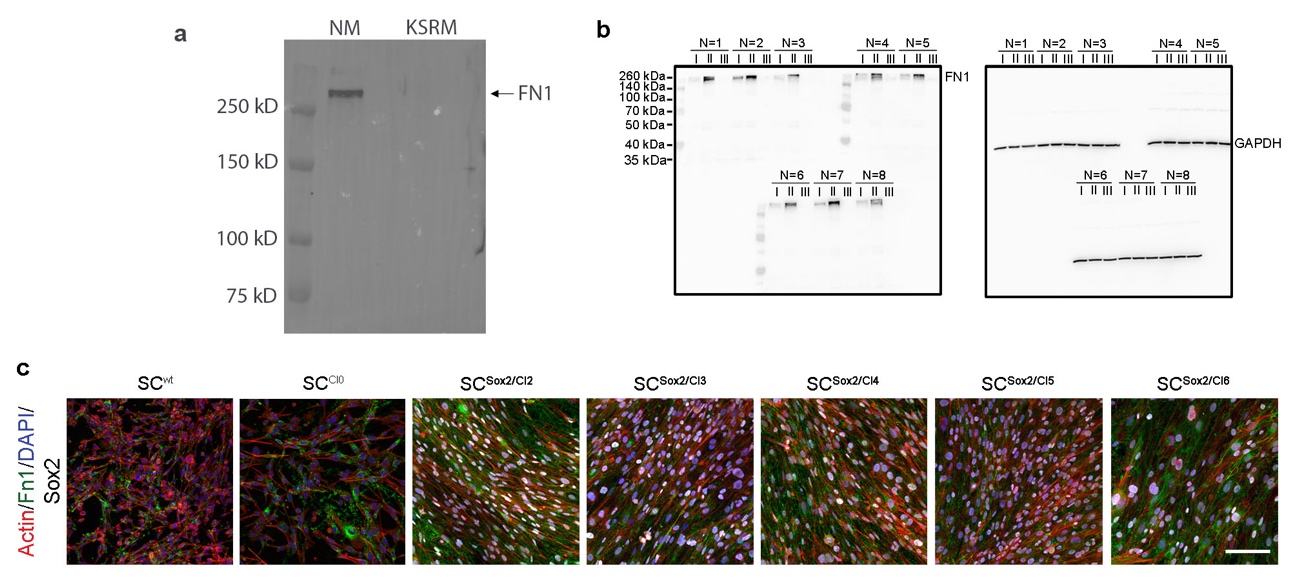
**

**Supplementary figure 3. FN expression in the Sox2-positive Schwann cell lines.** (a) Analysis of FN protein levels in 20μg of total protein of DMEM and KSRM supplemented with 10% FBS or KSR respectively, FN1 chemiluminescence image merged with the bright field image of the protein standard. (b) Western Blot analysis of FN1 protein levels in whole lysates of SC^wt^ (I), SC^Sox2/Cl2^ (II); and SC^Sox2/FnKO^ (III), GAPDH was used as loading control (N=8). (c) Representative immunostaining confocal images of SOX2 (grey), actin fibres (red), FN (green) of SC^wt^ and selected clones in FN-free KSR medium. Nuclei were counterstained with DAPI (blue). Scale bar, 100 μm.

**­
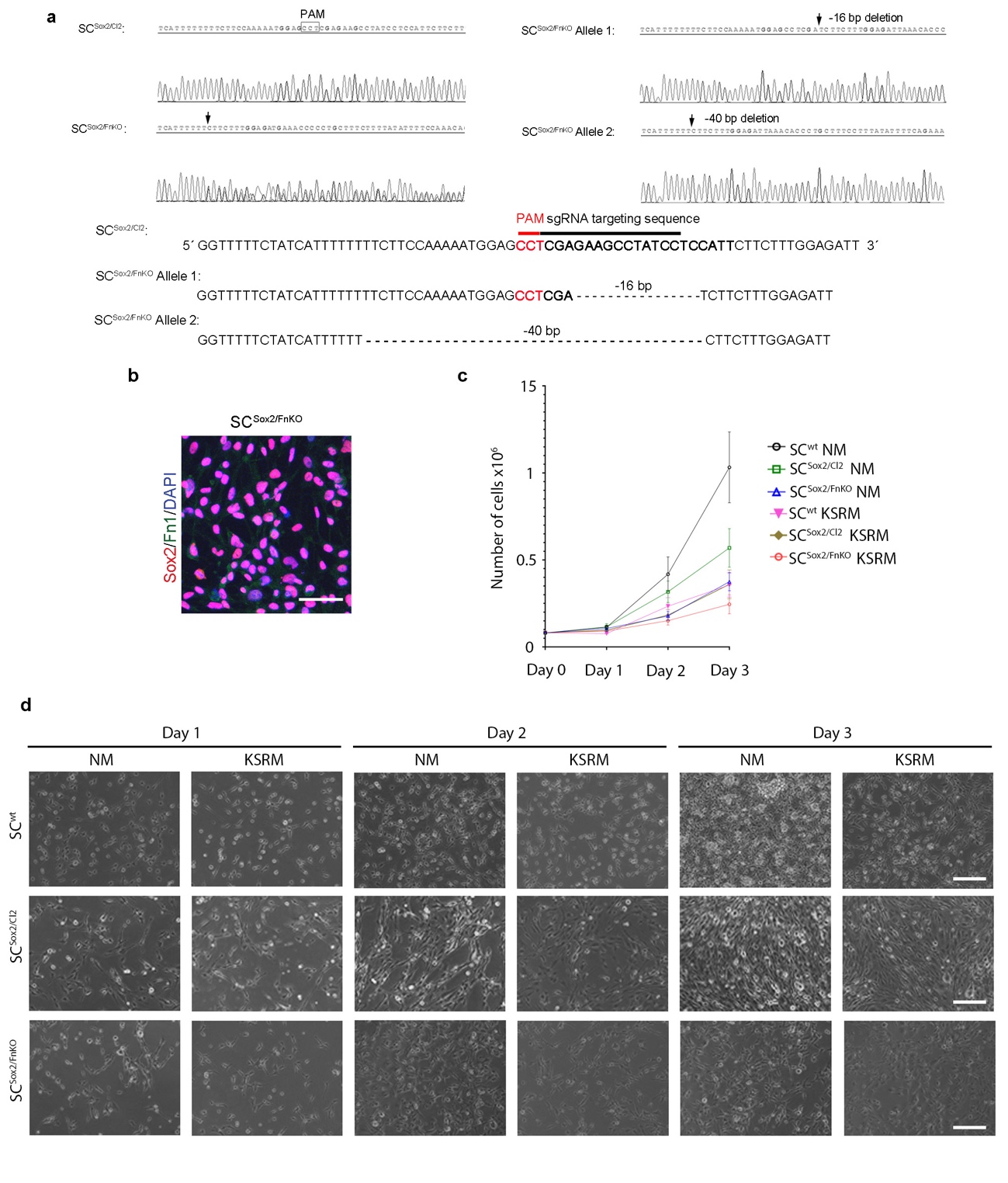
**

**Supplementary figure 4. Fibronectin expression in the different Sox2-positive cell clones and generation of a FN KO cell line from the SC^Sox2/Cl2^ using the CRISPR/Cas9 system.** (a and b) Sequencing readings of cloned PCR products of the *Fn1* gene from the SC^Sox2/FnKO^ cells, indicating the deletion of 16 bp and 40 bp within the sgRNA target region in each of the two alleles of the KO clone. (b) Representative confocal image of FN1 (green) and Sox2 (red) immunostaining of SC^Sox2/FnKO^. Nuclei were counterstained with DAPI (blue), scale bar 100 μm. (c and d) Total number of cells counted every 24 hours during 3 days. The graph shows the mean ± s.e.m. (N=5), a two-way ANOVA was used to evaluate the differences between cell lines cultured in the same medium or between the same cell line cultured in the two different media. (c) Representative phase contrast images of the Schwann cell lines cultured in NM and KSRM for 3 days, Scale bar, 200 μm.

**
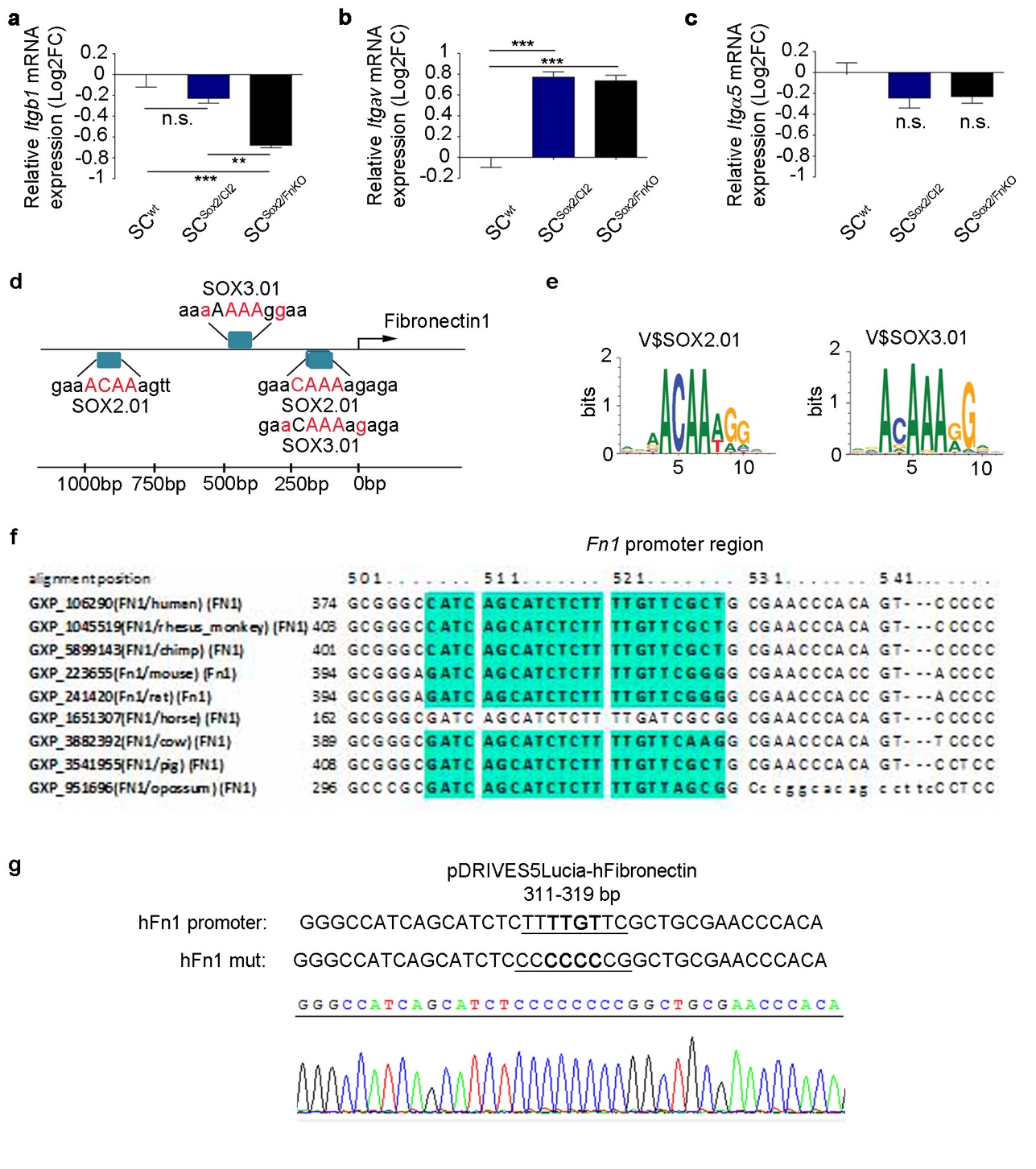
**

**Supplementary figure 5. Gene expression of integrins in Schwann cells and localization of Sox2 binding sites in the proximal promoter of FN.**  (a-c) Graphs show real-time RT-qPCR of mRNA expression in the SC^wt^, SC^Sox2/Cl2^, and SC^Sox2/FnKO^. Gene expression was normalized relative to *Gapdh, Ankrd27* and *Rictor*. One-way ANOVA (N=4). Graphs show mean value ± s.e.m, **p<0.005, ***p<0.0005. ***p<0.0005. n.s. Non-significant differences. (d) *In silico* promoter analysis of V$SORY family binding sites within the promoter sequences of the rat *Fn1* gene. Base pairs showed in red appear in a position where the matrix exhibits a high conservation profile, while capital letters denote the core sequence. (e) Representation of Sox2 and Sox3 binding sites, showing the similarity between the sequences recognized by the two transcription factors. (f) *in silico* promoter analysis of the V$SORY family shows a binding site in FN promoter which is conserved in different mammalian species. (g) Site-directed mutagenesis of Sox2 binding site in the human FN promoter cloned into the reporter plasmid pDRIVES5 Lucia used for the Lucia luciferase reporter assays. Image shows the substitution of 8 bp (underlined) of the Sox2 binding sequence (hFn1_mut).


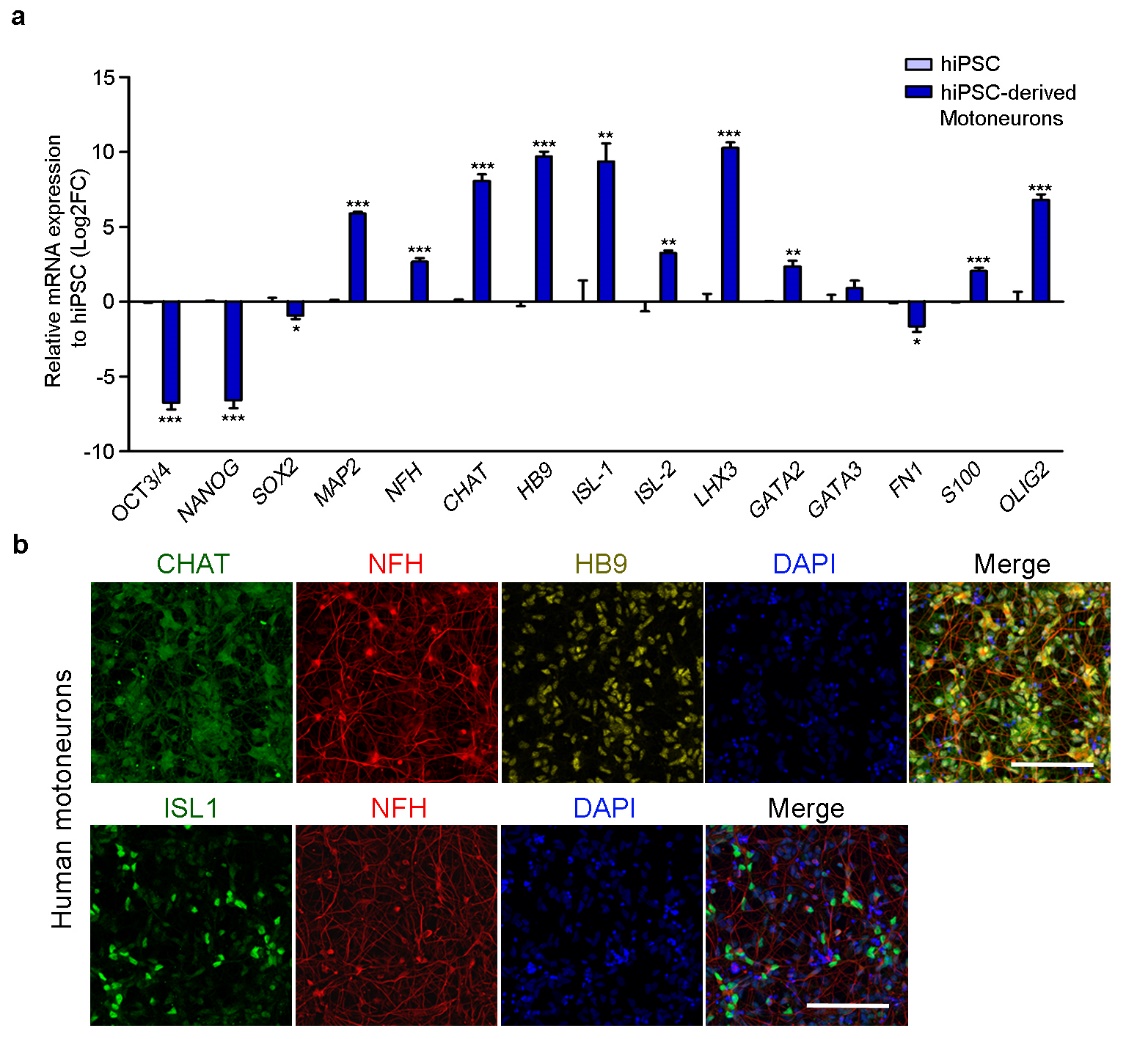


**Supplementary figure 6. Characterization of the hiPSC-derived motoneurons.** (a) Gene expression analysis of different markers of: motoneurons, stem cell and interneurons at the end of differentiation (day 21) of hiPSC-derived motoneurons and hiPSC as a control analysed by real-time RT-qPCR. *Gapdh, b-actin* and *18S* were used as a reference gene. t-test comparing motorneurons with hiPSC (N=4). (b) Immunostaining characterization of the human iPSC-derived motoneurons showing CHAT, NFH, and HB9 triple positive cells (top panel) and ISL1 and NFH double positive cell (bottom panel). Nuclei were counterstained with DAPI (blue). Scale bar, 50 μm. Results are shown as the mean ± s.e.m. *p<0.05, **p<0.005, ***p<0.0005.


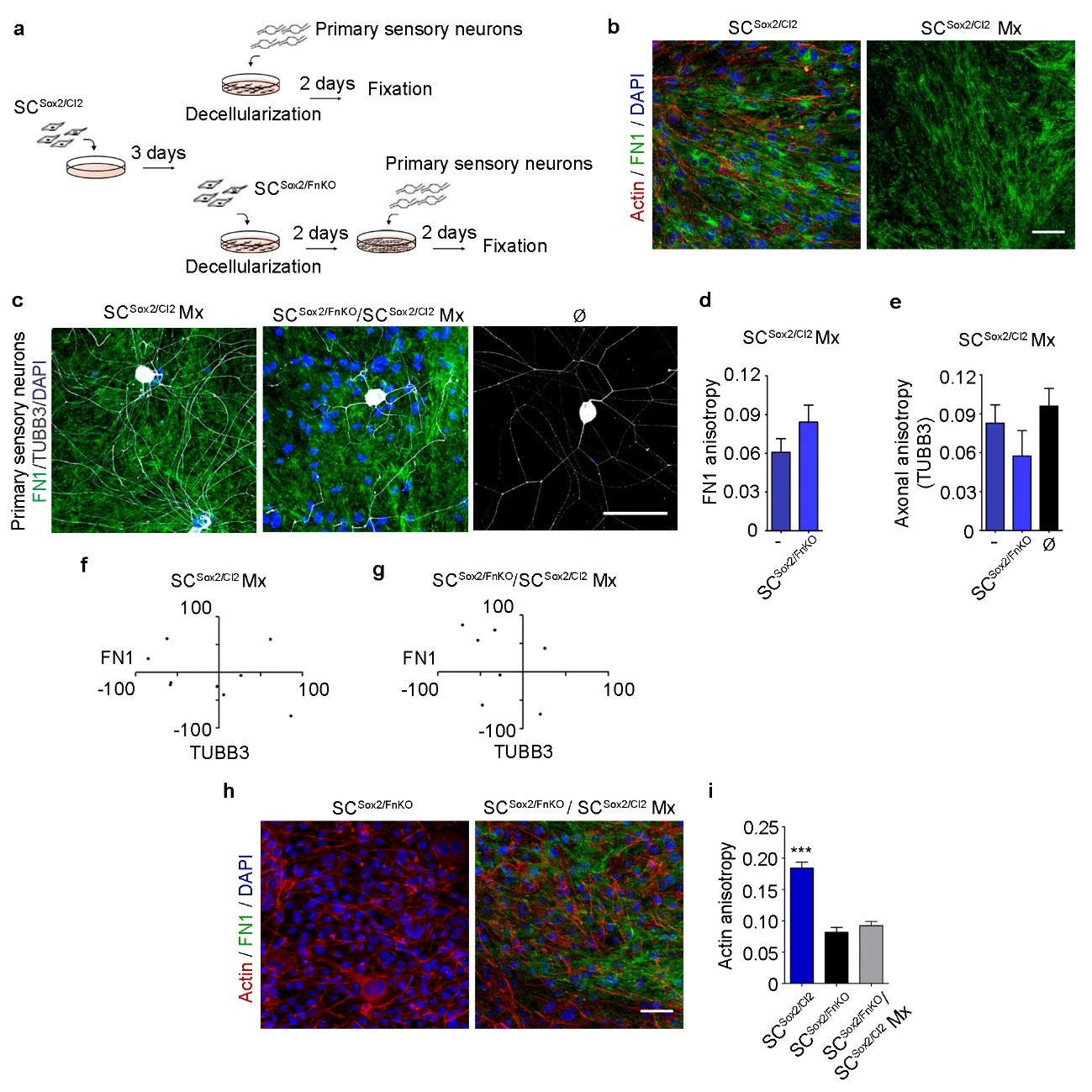


**Supplementary figure 7. Direct contact between neurons and Schwann cells is necessary for proper axon growth.** (a) Experimental design of co-culture between Schwann cells and mouse primary sensory neurons. (b) Representative confocal images of Actin fibres (red) and FN1 (green) immunostainings of SC^Sox2/Cl2^, SC^Sox2/Cl2^ matrix after the decellularization protocol. Nuclei were counterstained with DAPI. Scale bar 50 μm. (c) Representative confocal images of FN1 (green) and TUBB3 (white) immunostainings of primary sensory neurons co-cultured on the SC^Sox2/Cl2^ Mx and with SC^Sox2/FnKO^. Ø corresponds to primary sensory neurons cultured alone. Nuclei were counterstained with DAPI (blue). Scale bar, 100 μm. (d and e) Quantification of FN1 fibres and primary sensory neurons anisotropy cultured on the SC^Sox2/Cl2^ Mx (-) and with the SC^Sox2/FnKO^ (N=3, n≥7 areas). (f and g) Correlation of FN1 fibre angles of the SC^Sox2/Cl2^ Mx with the axon orientation in the absence and present of SC^Sox2/FnKO^ (TUBB3) (N=3). (h) Representative confocal images of Actin fibres (red) and FN1 (green) immunostainings of SC^Sox2/FnKO^, and SC^Sox2/FnKO^ cultured on the extracellular matrix generated by SC^Sox2/Cl2^. (i) Quantification of actin anisotropy of SC^Sox2/Cl2^, SC^Sox2/FnKO^, and SC^Sox2/FnKO^ cultured in the extracellular matrix generated by SC^Sox2/Cl2^. Graphs show mean value ± s.e.m, **p<0.005, ***p<0.0005. ***p<0.0005.


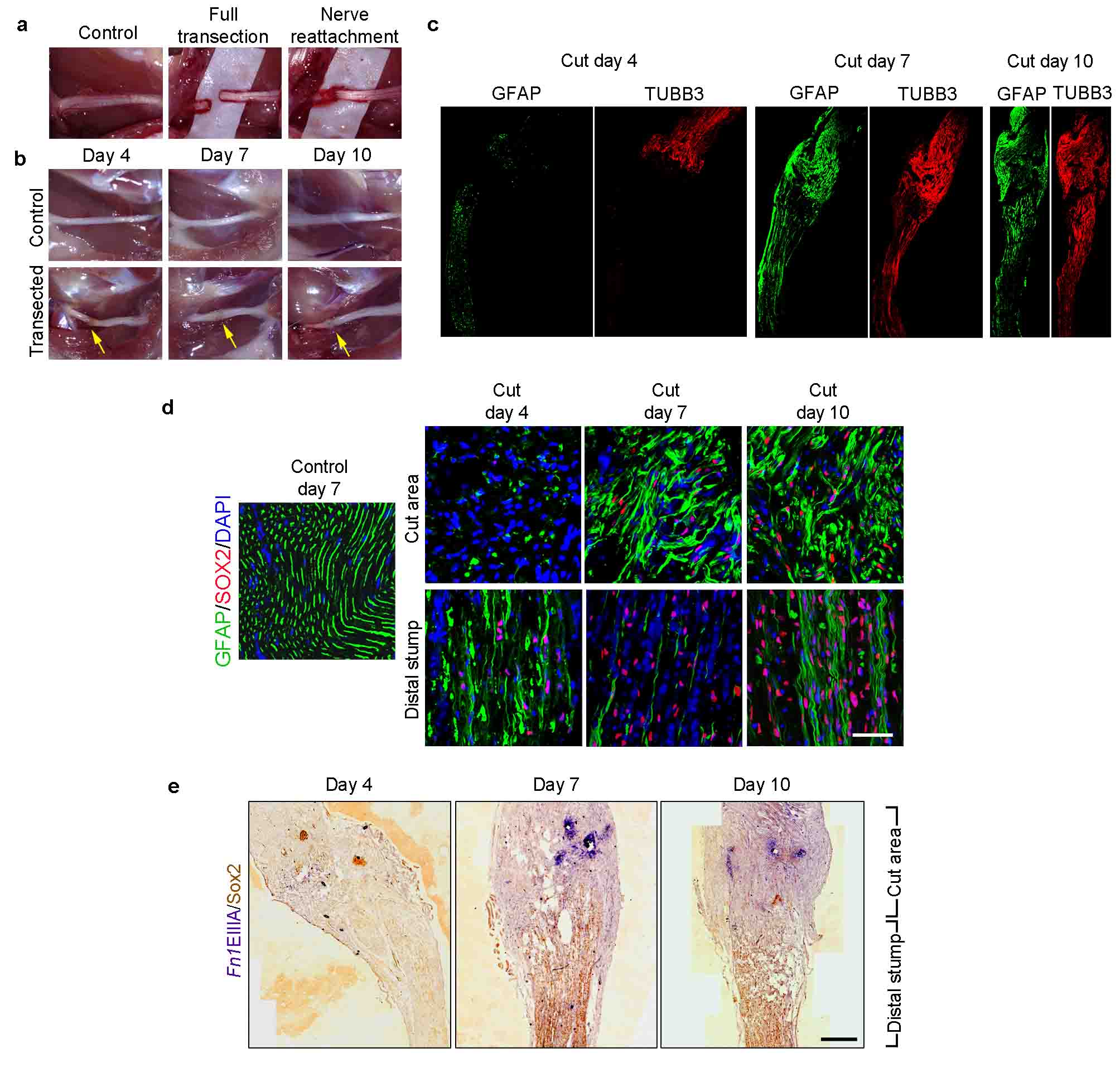


**Supplementary figure 8. Sox2 and FN are up-regulated after transection of rat sciatic nerve.** (a) Photographs of rat sciatic nerves before and after full transection followed by reattachment. (b) Photographs of the rat sciatic nerves at the different time points evaluated after surgery. (c) Representative confocal images of GFAP (green) and TUBB3 (red) immunostainings of sciatic nerve after complete transection at day 4, 7 and 10 post surgery. Nuclei were counterstained with DAPI (blue). (d) Representative confocal images of GFAP (green) and SOX2 (red) immunostainings of sciatic nerve in control condition (day 7) and after complete transection at day 4, 7 and 10 post surgery. Nuclei were counterstained with DAPI (blue). Scale bar, 50 μm. (e) *In situ* hybridization using a probe against *Fn1*EIIIA (purple) and SOX2 DAB staining (brown) in control and transected nerves at day 4, 7 and 10 post surgery showing the cut area and the distal stump. Scale bar, 500 μm. Results are shown as the mean ± s.e.m. *p<0.05.

**Table 1. Related to materials and methods.** List of antibodies used for immunostainings, Western Blotting and ChIP.

| Antibody | Company | Catalogue # |
| --- | --- | --- |
| Donkey anti-Rabbit IgH (H+L) Alexa Fluor 488 | Thermo Fisher | A-21206 |
| Donkey anti-Mouse IgH (H+L) Alexa Fluor 555 | Thermo Fisher | A-31570 |
| Donkey anti-Goat IgH (H+L) Alexa Fluor 555 | Thermo Fisher | A-21432 |
| Goat anti-Rabbit IgH (H+L) Alexa Fluor 633 | Thermo Fisher | A-21070 |
| Goat anti-Mouse IgH (H+L) Alexa Fluor 633 | Thermo Fisher | A-21052 |
| Alexa Fluor 568 Phalloidin | Thermo Fisher | A-12380 |
| Alexa Fluor 488 Phalloidin | Thermo Fisher | A-12379 |
| Anti-Choline Acetyltransferase (CHAT) | Abcam | ab18736 |
| Anti- Focal adhesion kinase (FAK) | Cell signalling | 3285 |
| Anti-phospho FAK (pFAK) | Invitrogen | 44-6246 |
| Anti-Fibronectin | DAKO | AO215 |
| Anti-GAPDH HRP | Sigma | G9295 |
| Anti-GAPDH | Cell Signaling | 2118S |
| Anti-GFAP | DAKO | ZO334 |
| Anti-GFAP (western blot) | Millipore-Sigma | G3893 |
| Anti-HB9 | Santa Cruz | sc-515769 |
| Anti-Histone3 (H3) | Cell signalling | 9715 |
| Anti-phophoHistone 3 | Millipore | 06-570 |
| Anti-Rabbit HRP | Jackson Labs | 11-035-003 |
| Anti-Mouse HRP | Sigma | A5278 |
| Anti-IgG (From MAGnify KIT) | Invitrogen | Module 3, 49-2024 |
| Anti-Islet 1 | Abcam | ab20670 |
| Anti-Neurofilament heavy polypeptide (NFH) | Abcam | ab4680 |
| Anti-Paxillin | Cell Signaling | 12065 |
| Anti-phopho Paxillin | Cell Signaling | 2541 |
| Anti-Sox2 (For immunocytochemistry) | Cell Signaling | 4900S |
| Anti-Sox2 (For ChIP) | Cell Signaling | 2748 |
| Anti-Sox2 (For immunohistochemistry) | Santa Cruz | sc-17320 |
| Anti-TUBB | Sigma | G9295 |
| Anti-TUBB3 | Biolegend | MMS-435P |
| Anti-Goat biotinylated | Vector | BA-5000 |
| DAPI | Thermo Fisher | D-1306 |
| Donkey anti-rabbit IRDye 680RD | LI-COR | 926-68073 |
| Donkey anti-mouse IRDye 800CW | LI-COR | 925-32212 |

**Table 2. Related to materials and methods.** List of primers used for real-time RT-qPCR.

| **Gene** | **Forward Primer** | **Reverse Primer** | **Specie** |
| --- | --- | --- | --- |
| *Sox2* | cgcagacctacatgaacg | gacttgaccacagagccc | Rat |
| *c-Jun* | gtgccaactcatgctaacg | tcgcaaccagtcaagttctc | Rat |
| *Gfap* | gctccaagatgaaaccaacc | tcatccgcctcctgtctg | Rat |
| *Krox20* | gcctgacagcctctaccc | gatcatgccatctccagcc | Rat |
| *Krox24* | acctgaccacagagtccttttc | gcaaccgggtagtttggct | Rat |
| *Fn1* | agacaaccatctcttggacg | ggaacttggaactgtaaggg | Rat |
| *Fn1EIIIA* | gcagtgaccaacattgacc | ccctgtacctggaaacttgc | Rat |
| *Fn1EIIIB* | attcacccgtttgctgtgtc | tggaacccggcattgactat | Rat |
| Adamts9 | agttcttagacactgggtatgga | gggaaggcaaaggataggt | Rat |
| *Lama5* | ttgtgctcgctccaagac | tgtgttgctgaactgcttctc | Rat |
| *Serpine* | agtagtgacaactgcaatcct | ggaacaagatggcacctg | Rat |
| *Plau* | gtaggaggagagttggttccc | aatacctcagctgttacacttcc | Rat |
| *Tgm2* | agaagagcgaaggaacatactg | tcatacttggtgctcaggtc | Rat |
| *Fbln1* | gccaggagtgtgctaacg | ctcatcaatatcttcgcaggtg | Rat |
| *Il1rl1* | catgatgtactcgacagtagatgg | agagctttgcagttcttaaacc | Rat |
| *Il33* | gagtatccaaggaacttcactg | tcacgtaacatccattctcca | Rat |
| *Apln* | ctttctaaagcaggattgaaggg | agatgagagccaaatagatgtgag | Rat |
| *Mmp10* | actcacattctccaggatctc | gtctccatgttctccaactg | Rat |
| *P1* ChIP | tctcaagctgttcaccac | ggcaaactcacaagacca | Rat |
| *P2* ChIP | ggtcacctttgacacgc | cgcctattcggtgttaggt | Rat |
| *P3* ChIP | gcaatgttctcaaacaccacc | gagccgactgaagcctg | Rat |
| *P4* ChIP | gaagttctccagtcccagacc | gggtacggtgggttcgc | Rat |
| Dist. Reg. ChIP | gtgtcagaaaccctgggaaa | ctcgcctccctctgtaactg | Rat |
| *Gapdh* | ccaccaactgcttagccccc | gcagtgatggcatggactgtgg | Rat |
| *Rictor** | gaggtggagaggacacaagccc | ggccacagaactcggaaacaagg | Rat |
| *Ankrd27** | cccaggatccgagaggtgctgtc | cagagccatatggacttcagggg | Rat |
| *OCT3/4*** | tctccaggttgcctctcact | gtggaggaagctgacaacaa | Human |
| *NANOG* | cagctacaaacaggtgaagacc | ccttctgcgtcacaccattg | Human |
| *SOX2* | ctcgcagacctacatgaacg | gaacccatggagccaagag | Human |
| *MAP2*** | ccgtgtggaccatggggctg | gtcgtcggggtgatgccacg | Human |
| *NFH* | cctaccaggaagccattcag | ccagagccatcttgacattgag | Human |
| *CHAT* | tcaagcacatgacgcagag | catttccaccgcagcctc | Human |
| *HB9* | cagttcaagctcaacaagtacc | gaaccaaatcttcacctgggtc | Human |
| *ISL-1* | tgcggagtgtaatcagtatttgg | tcccgtacaacctgatataatctc | Human |
| *ISL-2* | gactatgtcaggctgttcgg | actcgatgtggtacacgct | Human |
| *LHX3* | cgcttacaacacctcgcc | ggaaccaaacctgcacca | Human |
| *GATA2* | ctgttcagaaggccggga | cacaggcattgcacaggt | Human |
| *GATA3* | ctacccaggtgacccgag | cgactctgcaattctgcg | Human |
| *FN1* | ggcaactctgtcaacgaagg | acattcgttcccactcatctc | Human |
| *S100b* | acaaggaagaggatgtctgag | tgtctccctcccttccag | Human |
| *OLIG2* | ggcttcaagtcatcctcgtc | ctctgtcatttgcttcttgtcc | Human |
| *GAPDH*** | ttgaggtcaatgaaggggtc | gaaggtgaaggtcggagtca | Human |
| *b-ACTIN*** | ccttgcacatgccggag | gcacagagcctcgcctt | Human |
| *18S* | gaggatgaggtggaacgtgt | tcttcagtcgctccaggtct | Human |

Reference*^9^,**^10^

**Supplementary movie legends**

**Supplementary movie 1. SC^wt^ culture in maintenance medium. Related to Figure 1.** Time-lapse microscopy of wild-type Schwann cells. Frames were taken every 25 minutes for 72 hours.

**Supplementary movie 2. SC^Sox2/Cl2^ culture in maintenance medium. Related to Figure 1.** Time-lapse microscopy of Schwann cells overexpressing Sox2. Frames were taken every 25 minutes for 72 hours.

**Supplementary movie 3. SC^wt^ culture in maintenance medium. Related to Supplementary Fig. 1.** Time-lapse microscopy of wild-type Schwann cells. Frames were taken every 25 minutes for 4 hours. Cells were labelled with a cell tracker dye, number of cell in frame 1= 624.

**Supplementary movie 4. SC^Sox2/Cl2^ culture in maintenance medium. Related to Supplementary Fig. 1.** Time-lapse microscopy of Schwann cells overexpressing Sox2. Frames were taken every 25 minutes for 4 hours. Cells were labelled with a cell tracker dye, number of cell in frame 1= 577.

**Supplementary movie 5. SC^wt^ culture in KSR medium. Related to Figure 3.** Time-lapse microscopy of wild-type Schwann cells, in the absence of FN in the culture medium. Frames were taken every 25 minutes for 72 hours.

**Supplementary movie 6. SC^Sox2/Cl2^ culture in KSR medium. Related to Figure 3.** Time-lapse microscopy of Schwann cells overexpressing Sox2, in the absence of FN in the culture medium. Frames were taken every 25 minutes for 72 hours.

**Supplementary movie 7. SC^Sox2/FnKO^ culture in KSR medium. Related to Figure 3.** Time-lapse microscopy of Schwann cells overexpressing Sox2 and knockout for FN, in the absence of FN in the culture medium. Frames were taken every 25 minutes for 72 hours.

**Supplementary references**

1 Heigwer, F., Kerr, G. & Boutros, M. E-CRISP: fast CRISPR target site identification. *Nature methods* **11**, 122-123 (2014).

2 Prewitz, M. C. *et al.* Tightly anchored tissue-mimetic matrices as instructive stem cell microenvironments. *Nature methods* **10**, 788-794 (2013).

3 De Luca, A. C., Faroni, A. & Reid, A. J. Dorsal root ganglia neurons and differentiated adipose-derived stem cells: an in vitro co-culture model to study peripheral nerve regeneration. *JoVE (Journal of Visualized Experiments)*, e52543-e52543 (2015).

4 Diecke, S. *et al.* Novel codon-optimized mini-intronic plasmid for efficient, inexpensive, and xeno-free induction of pluripotency. *Scientific reports* **5** (2015).

5 Qu, Q. *et al.* High-efficiency motor neuron differentiation from human pluripotent stem cells and the function of Islet-1. *Nature communications* **5**, 3449, doi:10.1038/ncomms4449 (2014).

6 Xiao, Y. *et al.* High-resolution live imaging reveals axon-glia interactions during peripheral nerve injury and repair in zebrafish. *Disease Models and Mechanisms* **8**, 553-564, doi:10.1242/dmm.018184 (2015).

7 Pinto-Teixeira, F. *et al.* Inexhaustible hair-cell regeneration in young and aged zebrafish. *Biology Open* **4**, 903-909, doi:10.1242/bio.012112 (2015).

8 Boudaoud, A. *et al.* FibrilTool, an ImageJ plug-in to quantify fibrillar structures in raw microscopy images. *Nature protocols* **9**, 457-463 (2014).

9 Gambarotta, G. *et al.* Identification and validation of suitable housekeeping genes for normalizing quantitative real-time PCR assays in injured peripheral nerves. *PloS one* **9**, e105601, doi:10.1371/journal.pone.0105601 (2014).

10 Kirkeby, A. *et al.* Predictive markers guide differentiation to improve graft outcome in clinical translation of hESC-based therapy for Parkinson’s disease. *Cell stem cell* **20**, 135-148 (2017).
